# A cryptic long-chain 3-ketoacyl-ACP synthase in the *Pseudomonas putida* F1 unsaturated fatty acid synthesis pathway

**DOI:** 10.1016/j.jbc.2021.100920

**Published:** 2021-06-25

**Authors:** Huijuan Dong, Jincheng Ma, Qunyi Chen, Bo Chen, Lujie Liang, Yuling Liao, Yulu Song, Haihong Wang, John E. Cronan

**Affiliations:** 1 Guangdong Provincial Key Laboratory of Protein Function and Regulation in Agricultural Organisms, College of Life Sciences, South China Agricultural University, Guangzhou, Guangdong, China; 2 Department of Microbiology, University of Illinois at Urbana-Champaign, Urbana, Illinois, USA; 3 Department of Biochemistry, University of Illinois at Urbana-Champaign, Urbana, Illinois, USA

**Keywords:** fatty acid metabolism, fatty acid oxidation, fatty acid synthase (FAS), regulator, 3-ketoacyl-ACP synthase, repressor protein, ACP, acyl carrier protein, SFA, saturated fatty acid, UFA, unsaturated fatty acid

## Abstract

The *Pseudomonas putida* F1 genome contains five genes annotated as encoding 3-ketoacyl-acyl carrier protein (ACP) synthases. Four are annotated as encoding FabF (3-ketoacyl-ACP synthase II) proteins, and the fifth is annotated as encoding a FabB (3-ketoacyl-ACP synthase I) protein. Expression of one of the FabF proteins, FabF2, is cryptic in the native host and becomes physiologically important only when the repressor controlling *fabF2* transcription is inactivated. When derepressed, FabF2 can functionally replace FabB, and when expressed from a foreign promoter, had weak FabF activity. Complementation of *Escherichia coli fabB* and *fabF* mutant strains with high expression showed that *P. putida fabF1* restored *E. coli fabF* function, whereas *fabB* restored *E. coli fabB* function and *fabF2* restored the functions of both *E. coli fabF* and *fabB*. The *P. putida* Δ*fabF1* deletion strain was almost entirely defective in synthesis of *cis*-vaccenic acid, whereas the Δ*fabB* strain is an unsaturated fatty acid (UFA) auxotroph that accumulated high levels of spontaneous suppressors in the absence of UFA supplementation. This was due to increased expression of *fabF2* that bypasses loss of *fabB* because of the inactivation of the regulator, Pput_2425, encoded in the same operon as *fabF2*. Spontaneous suppressor accumulation was decreased by high levels of UFA supplementation, whereas competition by the *P. putida* β-oxidation pathway gave increased accumulation. The Δ*fabB ΔfabF2* strain is a stable UFA auxotroph indicating that suppressor accumulation requires FabF2 function. However, at low concentrations of UFA supplementation, the Δ*fabF2* ΔPput_2425 double-mutant strain still accumulated suppressors at low UFA concentrations.

*Pseudomonas putida* is a saprophytic soil γ-proteobacterium (1) that is readily distinguished from *Pseudomonas aeruginosa* by the lack of pyocyanin production and an inability to grow at 42 °C (2, 3). Although *P. putida* and *P. aeruginosa* have a high level of genomic conservation (85% of the predicted coding regions are shared), *P. putida* genomes lack key virulence factors including exotoxin A and type III secretion systems (4). Therefore, *P*. *putida* is considered a safe bacterium for cloning and expression of foreign genes and is a major *Pseudomonas* research organism (1, 5).

Fatty acids are the major components of membrane phospholipids (6). Bacteria use the type II fatty acid synthesis system to produce long-chain fatty acids through a cycle of elongation, reduction, dehydration, and reduction reactions catalyzed by a series of discrete enzymes (7) (Fig. S1).

The 3-ketoacyl-acyl carrier protein (ACP) synthases catalyze the elongation reactions of fatty acid synthesis (Scheme 1).

**Figure:**
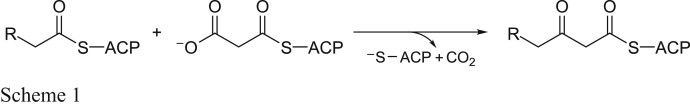

*Escherichia coli* has two long-chain 3-ketoacyl ACP synthases: 3-ketoacyl ACP synthase I (FabB) and 3-ketoacyl ACP synthase II (FabF). *E. coli* FabB is required to elongate *cis*-3-decenoyl ACP to long-chain unsaturated acyl-ACPs and together with FabA is a key enzyme in unsaturated fatty acid (UFA) synthesis (8). *E. coli* FabF is responsible for extending saturated acyl ACPs and for conversion of *cis*-9-hexadecenoyl-ACP (palmitoleoyl-ACP) to *cis*-11-octadecenoyl-ACP (*cis*-vaccenoyl-ACP), a reaction regulated by growth temperature (9). Although many diverse bacteria encode long-chain 3-ketoacyl ACP synthases similar to *E. coli* FabB and FabF, there are significant differences. The *Haemophilus influenzae* genome encodes only *fabB* and lacks *fabF* (10). *Clostridium acetobutylicium* and *Ralstonia solanacearum* each have only a single 3-ketoacyl ACP synthase, but these FabF enzymes have both 3-ketoacyl ACP synthetase I (FabB) and II (FabF) activities (11, 12). *Lactococcus lactis* FabF also has both FabB and FabF activities, but upon overexpression, the FabF can replace FabH, the short-chain 3-ketoacyl ACP synthase III (13). *Shewanella oneidensis* MR has one *fabB* and two *fabF* genes where *fabF1* has the functions of both 3-ketoacyl ACP synthase I and II (14). Increased expression of *fabF1* can restore the synthesis of UFAs when the pathway is blocked by inactivation of *fabB* (15).

The genome of *P. putida* F1 shows complexity in that four *fabF* genes are annotated: together with a single *fabB*, only the *fabF1* and *fabB* genes are located with other fatty acid synthesis genes (Fig. S2). Complementation studies using *E. coli fabB* and *fabB*(Ts) *fabF* mutant strains showed that *fabF2* has the functions of both *E. coli* enzymes when highly expressed. In the pseudomonads, *fabF2* is ubiquitous and homologous genes are found in other bacteria (see below). In this report, the function of *fabF2* and *fabB* in *P. putida* F1 was explored. Deletion mutants of *fabB* were unable to synthesize UFAs, although suppressors accumulated and accumulation required a functional *fabF2* gene. We report that Δ*fabB* suppression required increased expression of *fabF2* and this resulted from mutational activation of a repressor gene encoded in the same operon.

## Results

### *P. putida F1* FabF proteins and FabB protein functionally replace *E. coli* FabB and FabF *in vivo*

The *P. putida* F1 genome contains one gene annotated as *fabB*, Pput_1693, and four annotated as *fabF* genes: Pput_3798 (*fabF1*), Pput_2422 (*fabF2*), Pput_2974 (*fabF3*), and Pput_2975 (*fabF4*) (Fig. S2). Alignments of the protein sequences showed that the residue identities between FabB and FabF2 with *E. coli* FabB are 66.3% and 34.2%, respectively, whereas the residue identities between FabF1 and FabF2 and *E. coli* FabF are 66.9% and 45.7%, respectively. The FabF3 and FabF4 identities with *E. coli* FabF are 25.8% and 33.9%, respectively.

*E. coli* FabB and FabF are the canonical bacterial long-chain 3-ketoacyl-ACP synthetases and contain the characteristic Cys-His-His catalytic triad also found in the five putative *P. putida* 3-ketoacyl-ACP synthetases (Fig. S3). To test the function of these proteins in fatty acid biosynthesis, the encoding genes were inserted into the high copy number, arabinose-inducible vector pBAD24M, and the resulting plasmids were introduced into *E. coli* strains CY242 and CY244. The *E. coli fabB*(Ts) mutant strain CY242 lacks 3-ketoacyl-ACP synthase I activity at the nonpermissive temperature and is unable to grow at 42 °C (16), unless supplemented with a UFA (16). The *E. coli fabB*(Ts) *fabF* strain CY244 lacks 3-ketoacyl-ACP synthase II and 3-ketoacyl-ACP synthase I activity at the nonpermissive temperature. Owing to the lack of saturated fatty acid synthesis, strain CY244 is unable to grow at 42 °C even when supplemented with oleic acid (16, 17). The complementation studies showed that *fabB* and *fabF2* both restored growth of strains CY242 and CY244 at the nonpermissive temperature, whereas *fabF1* restored growth of CY244 (Fig. S4). Growth of the complemented strains was significantly inhibited by overexpression of *fabF1* at the nonpermissive temperature (Fig. S4). These data indicate that *P. putida* F1 FabB and FabF2 both have the activity of *E. coli* FabB, whereas FabF1 has the activity of *E. coli* FabF. Expression of *fabF3* and *fabF4* failed to either complement CY242 or CY244 (Fig. S4) or restore the fatty acid composition of the *△fabF1ΔfabF2* strain (Table S3), and we conclude that the encoded proteins lack 3-ketoacyl-ACP synthase activity.

**Figure 1:**
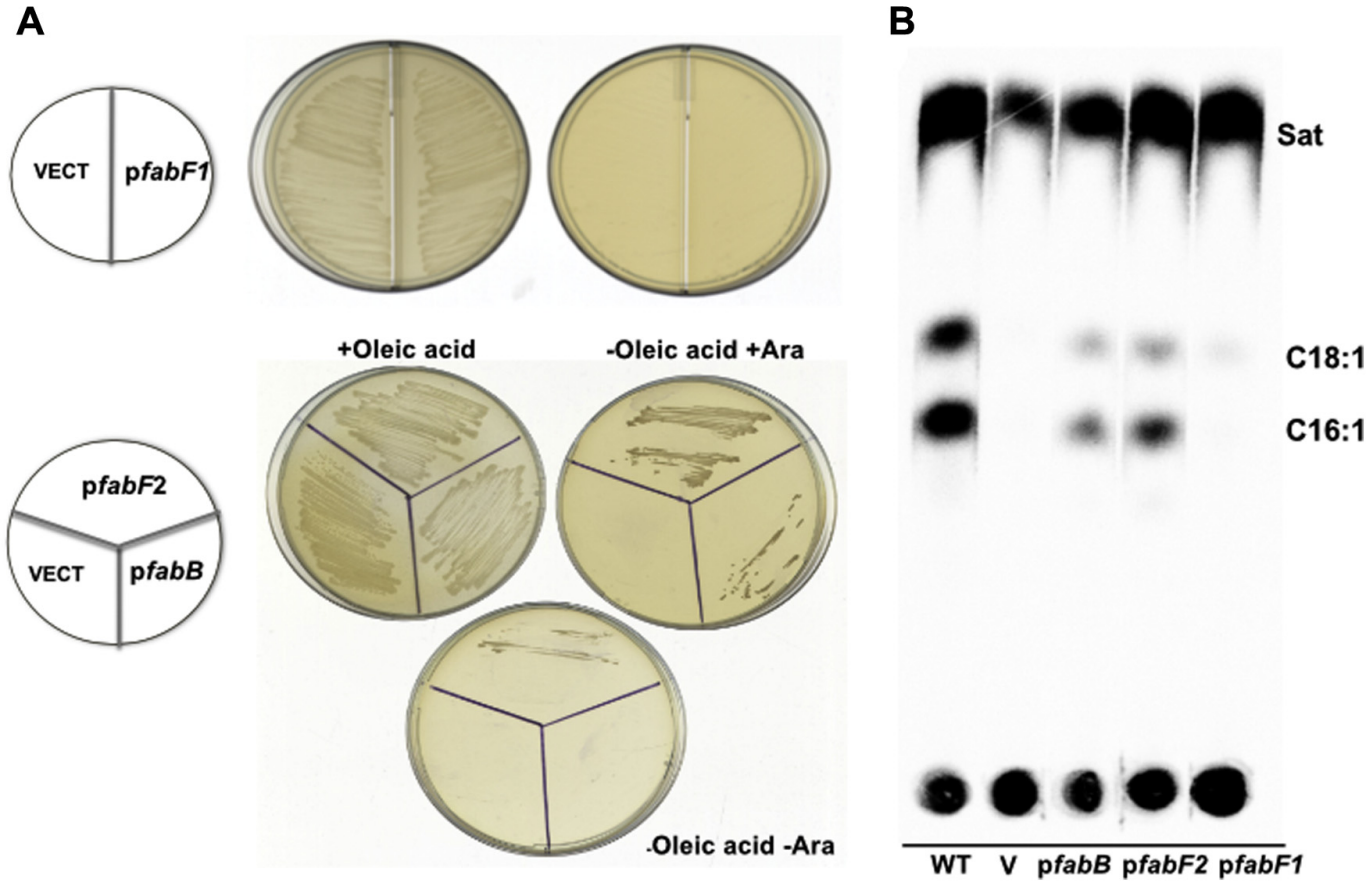
**Complementation of the *Escherichia coli fabB* strain K1060.***A*, growth of derivatives of *E. coli fabB* mutant strain K1060 expressing either *P. putida* F1 *fabF1*, *fabB*, *fabF2* or vector plasmids. The K1060 transformants were incubated on LB medium without or with 0.02% arabinose (Ara) or oleic acid (the *upper left* two plates contain oleate, whereas the *right* two plates lack oleate but contain arabinose). The single plate at the *bottom* lacks both oleate and arabinose. *B*, K1060 transformants were grown with [1-^14^C]acetate in LB medium with arabinose and analyzed by argentation TLC. UFA starvation of *E. coli* UFA auxotrophs does not result in immediate cessation of growth. Growth persists for at least a doubling as the phospholipid UFAs are diluted out. In panel *B*, the cells were washed thrice to remove oleate and then resuspended in the growth medium lacking oleate at the original cell density before labeling with [^14^C]acetate. WT is a WT *E. coli* strain, and V denotes the empty vector. TLC, thin layer chromatography; Vect, empty vector; UFA, unsaturated fatty acid.

*E. coli fabB* strain K1060 is auxotrophic for UFAs at all growth temperatures and was used to confirm the above results at the low temperatures where *P. putida* F1 grows best. Plasmids expressing *P. putida* F1 *fabB* or *fabF2* restored growth of strain K1060 in the absence of oleic acid (Fig. 1), further indicating that *P. putida fabB* and *fabF2* have *E. coli fabB* function. This was confirmed by labeling of cultures with [1-^14^C]acetate, which showed that both *fabB* and *fabF2* restored UFA synthesis to strain K1060 (Fig. 1). *E. coli* FabF is required for the elongation of *cis*-9-hexadecenoyl-ACP to *cis*-11-octadecenoyl-ACP (9, 12).

*E. coli* strain CL28 is a *ΔfabF* strain defective in synthesis of *cis*-11-octadecenoyl-ACP (Table S1). To test whether the *P. putida* F1 *fabFs* and *fabB* have *E. coli fabF* activity, the *P. putida* F1 *fabF* and *fabB* plasmids were transformed into strain CL28. The fatty acid compositions of the resulting strains were obtained by GC-MS. High levels of expression of either *P. putida* F1 *fabF1* or *fabF2* increased the *cis*-11-octadecenoate (*cis*-vaccenate) content of strain CL28 with *fabF1* expression giving the significantly greater response (Table 1). This indicated that *fabF1* and *fabF2* both have *E. coli fabF* function and can convert *cis*-9-hexadecenoyl-ACP to *cis*-11-octadecenoyl-ACP.

**Table 1:** Fatty acid compositions of CL28 complemented strains

Fatty acid %	CL28/vector	CL28/p*fabB*	CL28/p*fabF1*	CL28/p*fabF2*	CL28/p*fabF3*	CL28/p*fabF4*
C_14:0_	4.0 ± 0.1	5.0 ± 0.1	3.5 ± 0	4.3 ± 0.1	4.8 ± 0.5	44.3 ± 0.2
C_16:0_	27.0 ± 0.6	24.7 ± 0.2	29.2 ± 1.0	27.4 ± 1.1	25.7 ± 1.9	27.8 ± 0.7
C_16:1_	64.8 ± 0.4	67.0 ± 0.3	48.9 ± 2.1	61.7 ± 1.3	67.4 ± 2.0	65.0 ± 0.9
C_17:0 cyclo_	3.0 ± 0.1	1.4 ± 0.1	1.2 ± 0.1	2.1 ± 0.1	1.7 ± 0.4	2.0 ± 0.2
C_18:0_	0.3 ± 0.1	0.5 ± 0	2.0 ± 0.6	0.5 ± 0.1	0.3 ± 0.1	0.4 ± 0.1
C_18:1_	0.9 ± 0.3	1.3 ± 0.4	15.3 ± 1.7	4.1 ± 0.1	0.1 ± 0	0.3 ± 0.2

These experiments were done using a high copy number plasmid with transcription from the strong arabinose promoter and translation from a strong ribosome-binding site. The increase in C18:1 detected in the CL28/p*fabF2* strain is the product of high expression.

### Analysis of the *P. putida* F1 FabB and FabF2 activities *in vitro*

**Figure 2:**
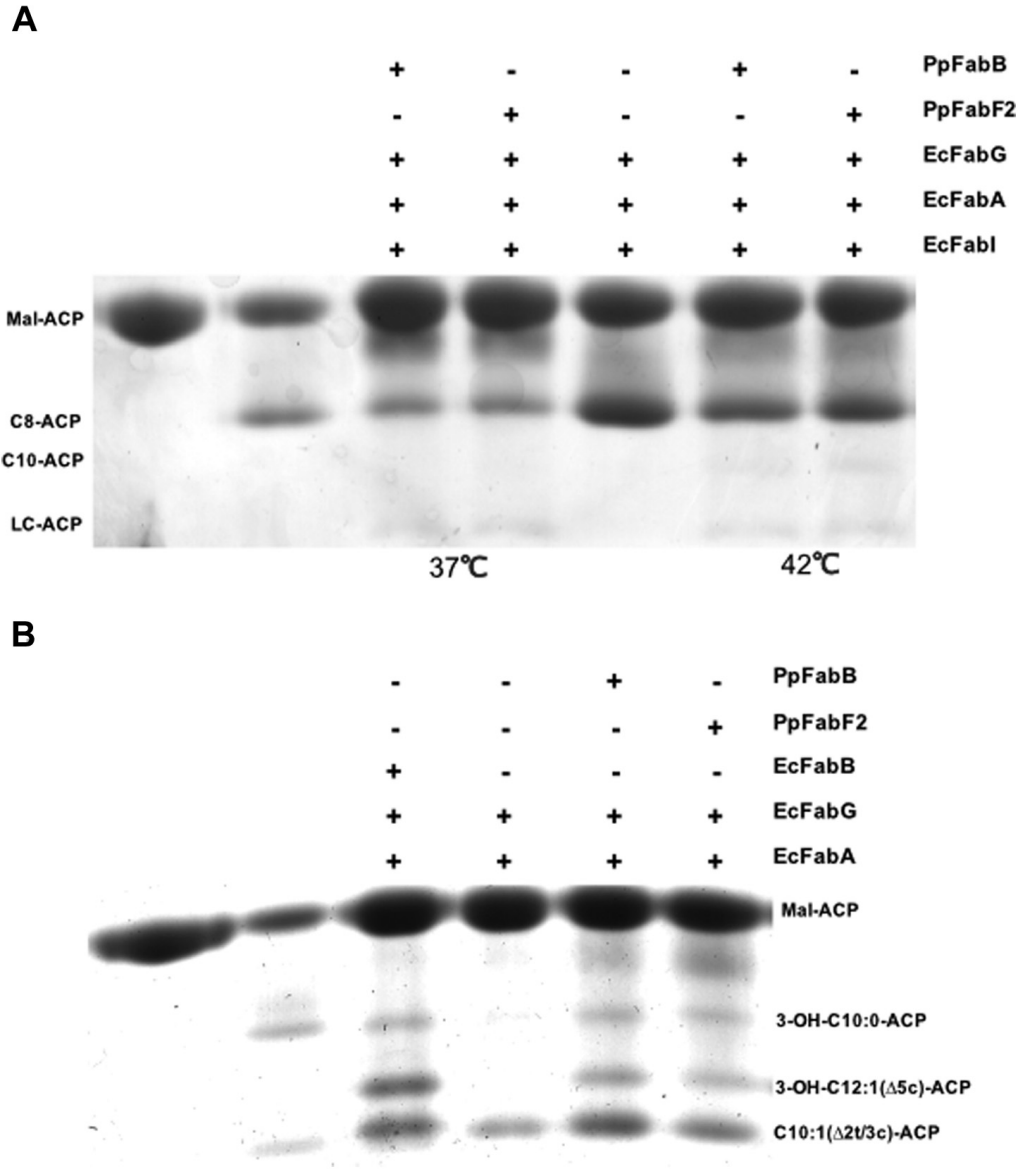
**Enzymatic activities of *Pseudomonas putida* FabB and FabF2 proteins *in vitro*.***A*, *P. putida* F1 FabB and FabF2 catalyze the extension of C8-ACP at 37 °C and 42 °C to long-chain (LC) acyl-ACP species. Note that the bands of the long-chain acyl-ACPs are faint. However, the lane lacking both *E. coli* FabB and FabF2 shows no long-chain species, whereas the lanes lacking either *E. coli* FabB or FabF2 show faint long-chain species bands. *B*, *P. putida* F1 FabB and FabF2 and *E. coli* FabB catalyze the extension of *cis*-3-decenoyl-ACP (denoted as C10:1 Δ2 or/Δ3-ACP) to 3-hydroxy-*cis*-5-dodecenoyl-ACP (denoted as 3-OH-C12:1(Δ5c)-ACP). Note that in the lane third from the right side that lacks all three 3-ketoacyl-ACP synthases, no products were formed. The 3-OH-C10:0-ACP is the product of hydration of *cis*-3-decenoyl-ACP by FabA (the three FabA products are in equilibrium). The first two lanes in the gels of both panels are standards. In both panels, malonyl-ACP is the *left*-most lane, whereas the next lane is the substrate for elongation. Each panel is a gel. ACP, acyl carrier protein.

The *P. putida* F1 FabB and FabF2 proteins were expressed in *E. coli* strain BL21(DE3) as described in Experimental procedures. The histidine-tagged fusion proteins were purified by nickel chelate chromatography. As measured by denaturing gel electrophoresis, the purified FabB and FabF2 proteins had monomeric molecular masses of 46 kDa consistent with the values calculated from the sequences of the tagged proteins (Fig. S5). Tryptic peptide mass spectral analyses confirmed the identities of the purified proteins (Fig. S5). To assay the activities of *P. putida* F1 FabB and FabF2, we used essentially the same methods to purify the *E. coli* fatty acid biosynthetic proteins, FabB, FabD, FabA, FabG, and FabI plus *Vibrio harveyi* acyl-ACP synthetase. *E. coli* holo-ACP was also purified. To test the functions of FabB and FabF2 *in vitro*, the elongation steps of the fatty acid synthesis reaction were reconstituted, followed by analysis by conformationally sensitive gel electrophoresis. At both 37 °C and 42 °C, *P. putida* F1 FabB and FabF2 elongate octanoyl-ACP using malonyl-ACP to generate low levels of long-chain acyl-ACP species (Fig. 2*A*). We also assayed the UFA synthetic abilities of purified PpFabB and PpFabF2 because this is their primary role in fatty acid synthesis. *E. coli* FabA converted 3-hydroxydecenoyl-ACP to a mixture of *trans*-2- and *cis*-3-decenoyl-ACPs. In the presence of *E. coli* FabB, *cis*-3-decenoyl-ACP is elongated with malonyl-ACP to 3-ketododecenoyl-ACP, which is reduced by *E. coli* FabG to give a new band corresponding to 3-hydroxy-*cis*-5-dodecenoyl-ACP. PpFabB and PpFabF2 both produce the same products as *E. coli* FabB (Fig. 2*B*) and hence are active in UFA synthesis *in vitro*.

### Construction of *P. putida* F1 fabF deletions and analysis of their phenotypes

**Figure 3:**
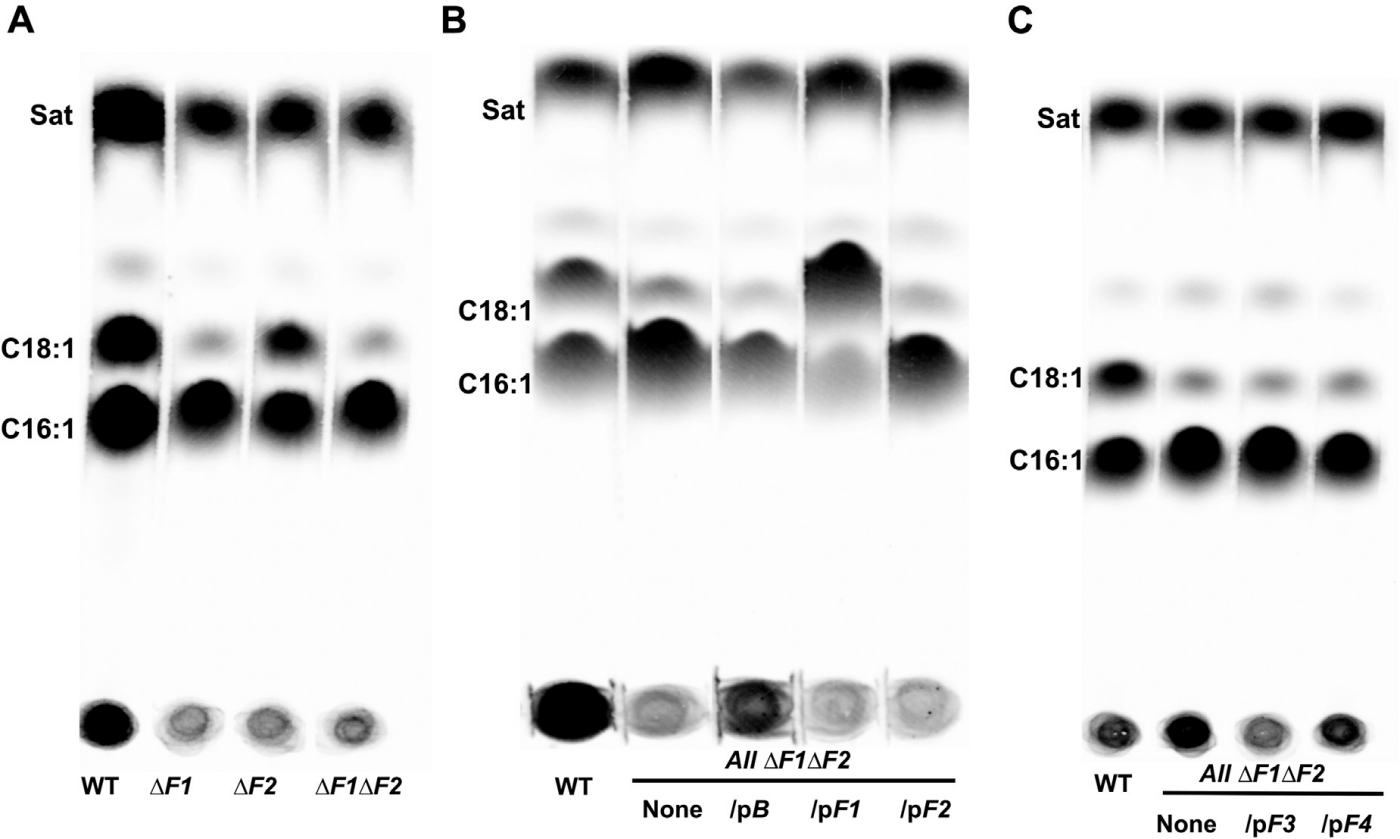
***De novo* fatty acid synthesis in the *Pseudomonas putida* F1 *ΔfabF1* and *ΔfabF2* strains plus the complemented strains.***A*, the phospholipid fatty acids of the WT, Δ*fabF1*, Δ*fabF2*, and Δ*fabF1 ΔfabF2* strains labeled with [1-^14^C]acetate. *B*, strains carrying plasmids encoding *fabB*, *fabF1*, or *fabF2* were labeled as above; the *fabF1* plasmid restored C18:1 synthesis to the Δ*fabF1 ΔfabF2* strain. In panel *B*, the host strain in the right-hand four lanes was the *ΔfabF1 fabF2* double mutant (none denotes the absence of a plasmid). In panel *C*, the host strain in the right-hand three lanes was the *ΔfabF1 fabF2* double mutant (none denotes the absence of a plasmid). Strains carrying plasmids encoding *fabF3* or *fabF4* were labeled as above and showed no activity. The figure is composed of scans of three different autoradiograms of the thin layer plates used to separate the radioactive methyl ester species. The panels are scans of autoradiograms of the three thin layer plates used to separate the radioactive methyl ester species. Each panel is from a separate thin layer plate.

To determine the physiological functions of the *P. putida* F1 *fabF* proteins in fatty acid biosynthesis, strains having each *fabF* gene deleted were constructed by allelic replacement. The fatty acid compositions of the *ΔfabF1* and *ΔfabF2* strains were determined by [1-^14^C]acetate labeling (Fig. 3) and GC-MS (Table 2). Relative to the WT strain, the Δ*fabF1* strain showed a large decrease in *cis*-vaccenic acid (C18:1), with a concomitant increase in palmitoleic acid (C16:1). The fatty acid compositions of the *fabF2*, *fabF3*, and *fabF4* deletion strains were indistinguishable from that of the WT strain. The fatty acid composition of the Δ*fabF1 ΔfabF2* double mutant was essentially the same as that of the Δ*fabF1* strain (Table S3).

**Table 2:** Fatty acid compositions of *Pseudomonas putida* F1 *fabF* strains

Fatty acid %	WT	*ΔfabF1*	*ΔfabF2*	*ΔfabF1 ΔfabF2*	*ΔfabF3*	*ΔfabF4*
C_16:0_	20.8 ± 3.0	18.6 ± 0.6	16.3 ± 5.5	18.9 ± 0.6	18.0 ± 0.6	19.6 ± 1.7
C_16:1_	49.4 ± 4.0	77.0 ± 0.4	47.6 ± 5.6	76.1 ± 1.1	47.3 ± 3.2	48.2 ± 4.0
C_18:0_	0.5 ± 0.2	0.2 ± 0.1	0.2 ± 0.1	0.3 ± 0	1.2 ± 0.3	0.6 ± 0.1
C_18:1_	29.2 ± 1.6	4.1 ± 0.7	34.9 ± 0.6	4.7 ± 0.5	34.0 ± 1.3	31.5 ± 2.2

The *ΔfabF1* and *ΔfabF2* strains plus the *ΔfabF1 ΔfabF2* double-mutant strain were complemented with plasmids carrying the WT copy of a gene, and the fatty acid compositions of the strains were determined by GC-MS. In the *fabF1* complemented strain, the levels of C18:1 sharply increased and the C16:1 level sharply decreased (Table S3). Hence, *P. putida* F1 FabF1 is responsible for saturated fatty acid (SFA) synthesis and for conversion of C16:1-ACP to C18:1-ACP. Expression of *fabF3* or *fabF4* failed to remedy defects in C18:1 synthesis because of loss of *fabF1*, whereas *fabF*2 had only weak activity (Table S3).

### Construction and properties of the *P. putida* ΔfabB strain

**Figure 4:**
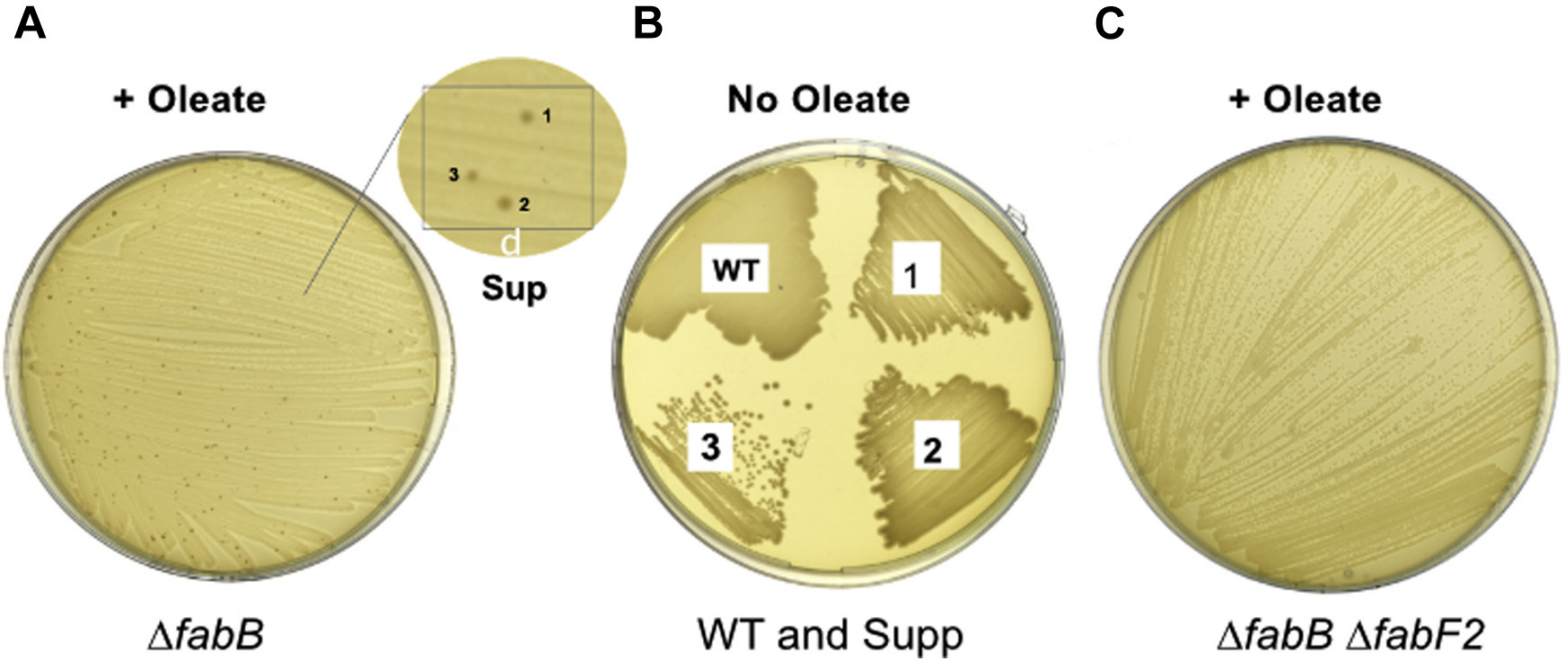
**Characterization of Δ*fabB* and Δ*fabB ΔfabF2* mutants of *Pseudomonas putida*.***A*, the Δ*fabB* strain was a UFA auxotroph and suppressors (1–3) (denoted as Supp) accumulated on LB medium plates containing oleic acid (see *inset*). *B*, upon restreaking, the suppressors restored growth on LB plates lacking oleic acid. *C*, the Δ*fabB* Δ*fabF2* strain was stably auxotrophic and did not accumulate suppressors.

A *P. putida ΔfabB* mutant strain was constructed by replacing the gene with a kanamycin resistance marker. The resulting Δ*fabB* mutant strain was a UFA (oleic acid) auxotroph demonstrating the importance of the gene in *P. putida* F1 UFA synthesis (Fig. 4*A*). However, the Δ*fabB* strain was unstable and spontaneously accumulated suppressors even at high oleate concentrations. This argued that a mutation that allowed the *ΔfabB* strain to synthesize UFA gave faster growth than that obtained by oleate supplementation. The dependence on oleate concentration argued that most of the oleate was consumed by β-oxidation, thereby limiting the amount available for phospholipid synthesis. Endogenous synthesis avoids β-oxidation because ACP rather than CoA thioesters are used and the 3-hydroxy thioester intermediates use opposite stereoisomers. The Δ*fabB* suppressor mutations restored UFA synthesis and allowed growth without oleate (Fig. 4*B*). The above data indicated that FabF2 has the functions of both *E coli* FabB and FabF, so it seemed likely that some alteration of *fabF2* could explain the accumulation of suppressors. To test this possibility, a Δ*fabB* Δ*fabF2* double-mutant strain was constructed. The Δ*fabB* Δ*fabF2* strain was a UFA auxotroph that failed to form suppressors (Fig. 4*C*). This indicated that a mechanism requiring *fabF2* could replace *fabB* function.

**Figure 5:**
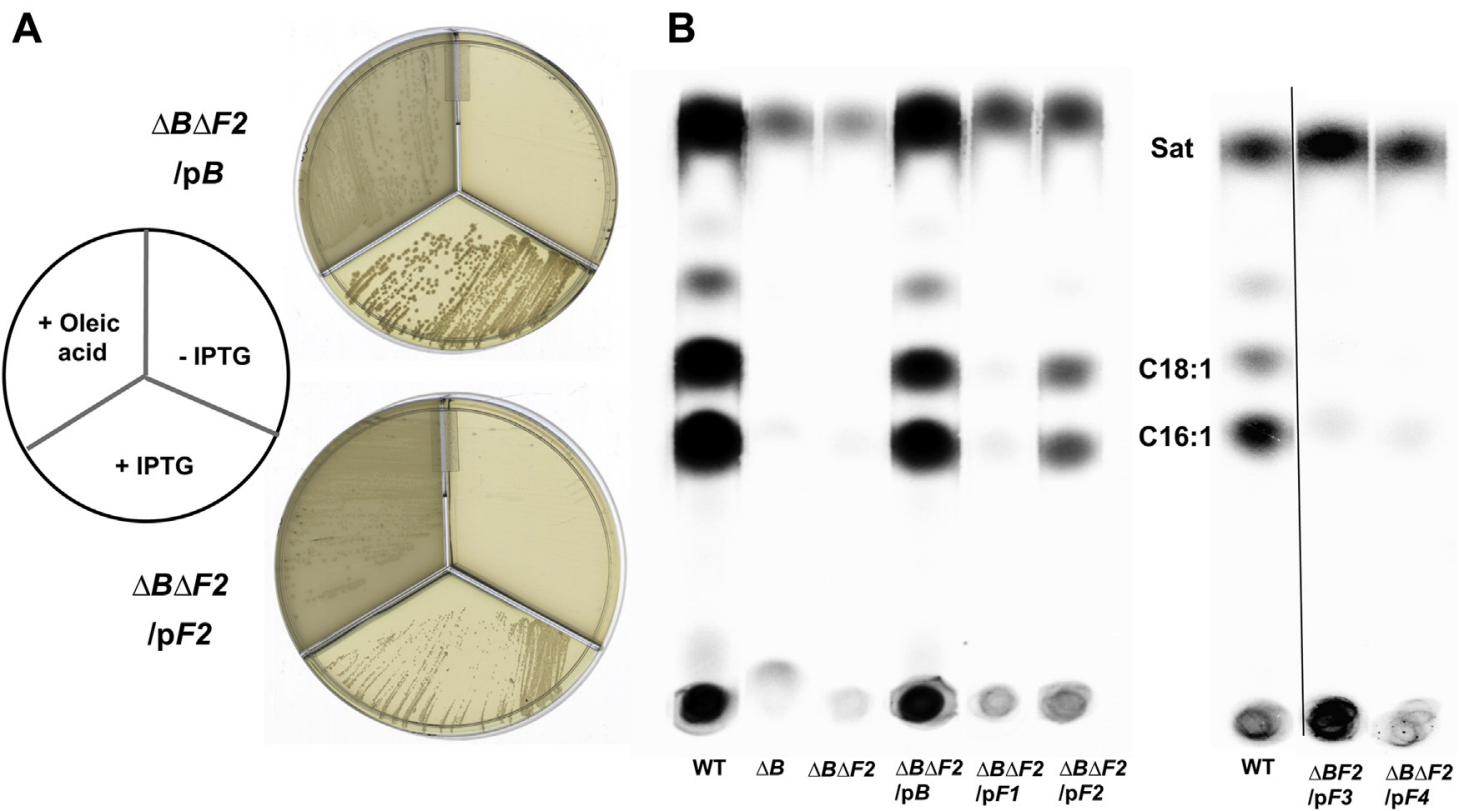
**Either the *fabB* or *fabF2* genes of *Pseudomonas putida* F1 complement the *ΔfabB ΔfabF2* double mutant.***A*, both *fabB* and *fabF2* restore the growth of *ΔfabB ΔfabF2* mutant in the absence of oleic acid. The growth of the *fabB* complemented strain is significantly better than that of the *fabF* complemented strain upon induction with 1 mM IPTG. *B*, the phospholipid fatty acids of *P. putida* F1 WT *ΔfabB*, *ΔfabB ΔfabF2*, and the complemented strains labeled with [1-^14^C]acetate (*ΔfabB* (*ΔB*), *ΔfabB ΔfabF2* (*ΔBF2*)) lack UFA synthesis. These are scans of autoradiograms of two different thin layer chromatographic separations. In the right-hand autoradiogram, an erroneously loaded lane was to the *right* of the first lane. This lane was deleted, and the remaining lanes spliced together. The splice junction is depicted by the *vertical line*. The right-hand three lanes are from a separate thin layer. *ΔBF2*/p*B*, *fabB* plasmid complementation of the *ΔfabB ΔfabF2* strain, *ΔBF2*/p*F*, *fabF* plasmid complementation of the *ΔfabB ΔfabF2* strain.

Plasmids encoding *fabF2* or *fabB* complemented the Δ*fabB* Δ*fabF2* strain (Fig. 5). The complemented strains restored growth and UFA synthesis and no longer required oleic acid. The growth phenotypes of the complemented strains indicated that FabB was more active than FabF2 (Fig. 5*A*). We also tested plasmids encoding *fabF1*, *fabF3*, and *fabF4* for the ability to restore UFA synthesis in the Δ*fabB* Δ*fabF2* strain by [1-^14^C]acetate labeling (Fig. 5*B*) and found no role for these genes in *P. putida* UFA synthesis *per se* although FabF1 can elongate 16:1 to 18:1 (Table 1). The failure of *fabF2* to complement the *P. putida* Δ*fabF1* strain, although it complements the *E. coli ΔfabF* strain, seems likely to be poor expression from the pSRK vector promoter in *P. putida* plus the high levels of expression in *E. coli*. Note that upon FabB overexpression, significant levels of *cis*-vaccenate are synthesized in a *fabF* strain (17).

*P. aeruginosa* PAO1 has three pathways for UFA synthesis (18), whereas *P. putida* F1 has only two pathways: the *fabA-fabB* pathway and the *desA* desaturase. Although the *P. putida* desaturase (Pput_0232) has 84% identity to the *P. aeruginosa* DesA protein, it is unable to support growth of a *ΔfabB* strain, although it may be responsible for the traces of UFAs seen in the *ΔfabB ΔfabF2* strain. To further test the ability of *P. putida* FabF2 to convert C16:1-ACP to C18:1-ACP, a Δ*fabF1* Δ*fabF2* Δ*desA* triple deletion strain was constructed by homologous recombination. In this triple mutant, the chromosomal *fabB* gene was replaced by *fabF2* (Fig. S6). Therefore, FabF2 was the sole long-chain 3-ketoacyl-ACP synthase in this strain. Fatty acid compositions obtained by [1-^14^C]acetate labeling (Fig. S6) and GC-MS (Table S4) showed that the level of C18:1 increased only slightly (4.4%–6.4%) accompanied by a moderate decrease in the C16:1 level (69.3%–63.4%) relative to the Δ*fabF1* Δ*fabF2* Δ*desA* triple mutant. Hence, in the native host, FabF2 has only a weak ability to convert of C16:1-ACP to C18:1-ACP.

### Analysis of suppressor phenotypes: Interaction with the *β*-oxidation pathway and inhibition by octanoic acid

**Figure 6:**
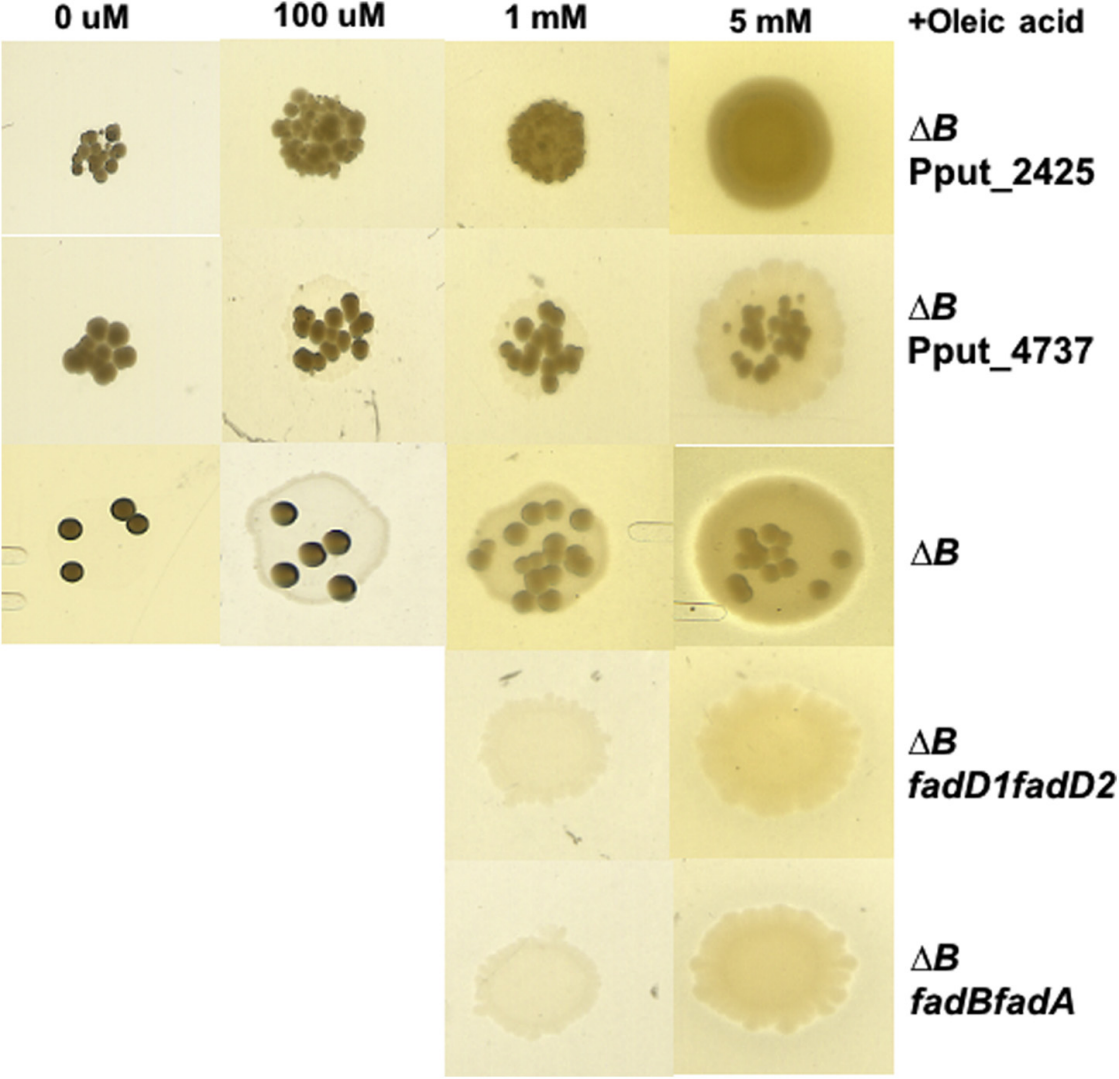
**Analysis of the growth phenotypes of *fabB* mutants in different concentrations of oleic acid and dependence on β-oxidation.** The *ΔfabB Δ*Pput_2425 strain (denoted as *ΔBΔ*Pput_2425) grew stably with 5 mM oleic acid, but as the concentration of oleic acid decreased, suppressors accumulated. The *ΔfabB Δ*Pput_4737 strain denoted by *ΔBΔ*Pput_4737 and the *ΔfabB* strain denoted by *ΔB* both accumulated suppressors at the lower concentrations of oleic acid. The *ΔfabB ΔfadD1 ΔfadD2* and *ΔfabB ΔfadB ΔfadA* strains (denoted as *ΔfabB ΔfadD1 ΔfadD2* and *ΔBfadBfadA*, respectively) grew stably at the high oleic acid concentration.

A high concentration of oleic acid (5 mM) generally decreased the accumulation of Δ*fabB* suppressors relative to lower concentrations, suggesting that the suppressor accumulation is related to the concentration of oleic acid (Fig. 6). After entry into the cell, oleic acid becomes activated by an acyl-CoA synthetase (FadD), and most of the acyl-CoA will enter the *β*-oxidation cycle for degradation to acetyl-CoA. Thus, only a small fraction of the UFA is available for synthesis of membrane phospholipids.

FadD1 and FadD2, which are homologs of the *P. aeruginosa fadD1* and *fadD2* acyl-CoA synthetase genes, have been identified as the major acyl-CoA synthetases in *P. putida* (18, 19). However, the *ΔfadD1 ΔfadD2* double mutant remains able to grow with oleic acid as the sole carbon source in minimal medium (20, 21), indicating the presence of other fatty acid activation pathways. We inactivated both *fadD* genes in a *ΔfabB* strain and found that oleic acid supplementation remained effective indicating that, as in *P. aeruginosa* (20), activation systems other than FadD1 and FadD2 are present in *P. putida* F1. Alignments with *E. coli* FadB and FadA indicated that Pput_3606 and Pput_3605 encoded β-oxidation cycle enzymes: enoyl-CoA hydratase/3-hydroxyacyl-CoA dehydrogenase (FadB) and 3-ketoacyl-CoA thiolase (FadA), respectively. Inactivation of the *β*-oxidation cycle enzymes in the Δ*fabB* strain blocked accumulation of suppressors as did inactivation of FadD1 and FadD2 (Fig. 6), indicating that accumulation of Δ*fabB* suppressors is closely related to the fatty acid *β*-oxidation pathway. Degradation of most of the oleate supplements limits the supply of oleoyl-CoA available for incorporation into phospholipids and provides a strong selection for accumulation of suppressors. The phenotype of the Δ*fabB ΔfadA ΔfadB* triple mutant was as expected, whereas the Δ*fabB ΔfadD1 ΔfadD2* triple mutant argues that the product of the unknown pathway that activates oleate in this strain somehow avoids *β*-oxidation. Note that the Δ*fabB ΔfadD1 ΔfadD2* and Δ*fabB ΔfadA ΔfadB* triple mutants grew relatively slowly, arguing that *P. putida* F1 derives significant carbon and energy from β-oxidation even when grown in a rich medium (Fig. 6). We first suspected that mutations in the *fabF2* coding sequence or the upstream promoter region were responsible for suppression of the *ΔfabB* mutant strain. However, sequencing showed that this was not the case. We then considered Pput_2425, a gene annotated as encoding a putative regulatory protein of the TetR family, which is the first gene in the *fabF2* operon (*fabF2* is the last gene, the two intervening genes encode an unrelated efflux pump) (Fig. S2). Pput_2425 seemed a good candidate because it appeared to be cotranscribed with *fabF2* and several members of the TetR family participate in regulation of fatty acid synthesis (22, 23). To test if the protein encoded by Pput_2425 is involved in *ΔfabB* suppression, we constructed a Δ*fabB* ΔPput_2425 double-mutant strain. The double mutant grew stably in LB medium containing 5 mM oleic acid and weakly in the absence of oleic acid with accumulation of suppressors. Given these data, we selected several independent suppressor strains and sequenced their Pput_2425 genes plus the upstream regions and found that all suppressors had mutations within the coding sequence. Five had deletion mutations (two 299(A), 265(C), 50–51(AT), 159(C)) and one had a missense mutation 130(G-C). The frameshifts caused by the 50 to 51(AT), 299(A), and 265(C), respectively, gave extended out-of-frame translation products of 18, 19, and five residues before a termination codon was encountered, whereas the 159(C) mutation directly created a TAA termination codon. These data together with the deletion allele indicate that the regulatory protein encoded by Pput_2425 is a repressor that negatively regulates *fabF2* expression. Note that deletion of the putative exporter genes, Pput_2424 and Pput_2423, in the *ΔfabB* strain failed to alter the phenotype of the *ΔfabB* strain. These strains remained auxotrophic and accumulated suppressors (data not shown). To test the possible influence of transcriptional polarity, an in-frame deletion allele of Pput_2425 was constructed in the *ΔfabB* strain. This strain accumulated suppressors in the absence of oleic acid as previously seen for the original *ΔfabB* strain that contained the prior Pput_2425 deletion allele (data not shown).

**Figure 7:**
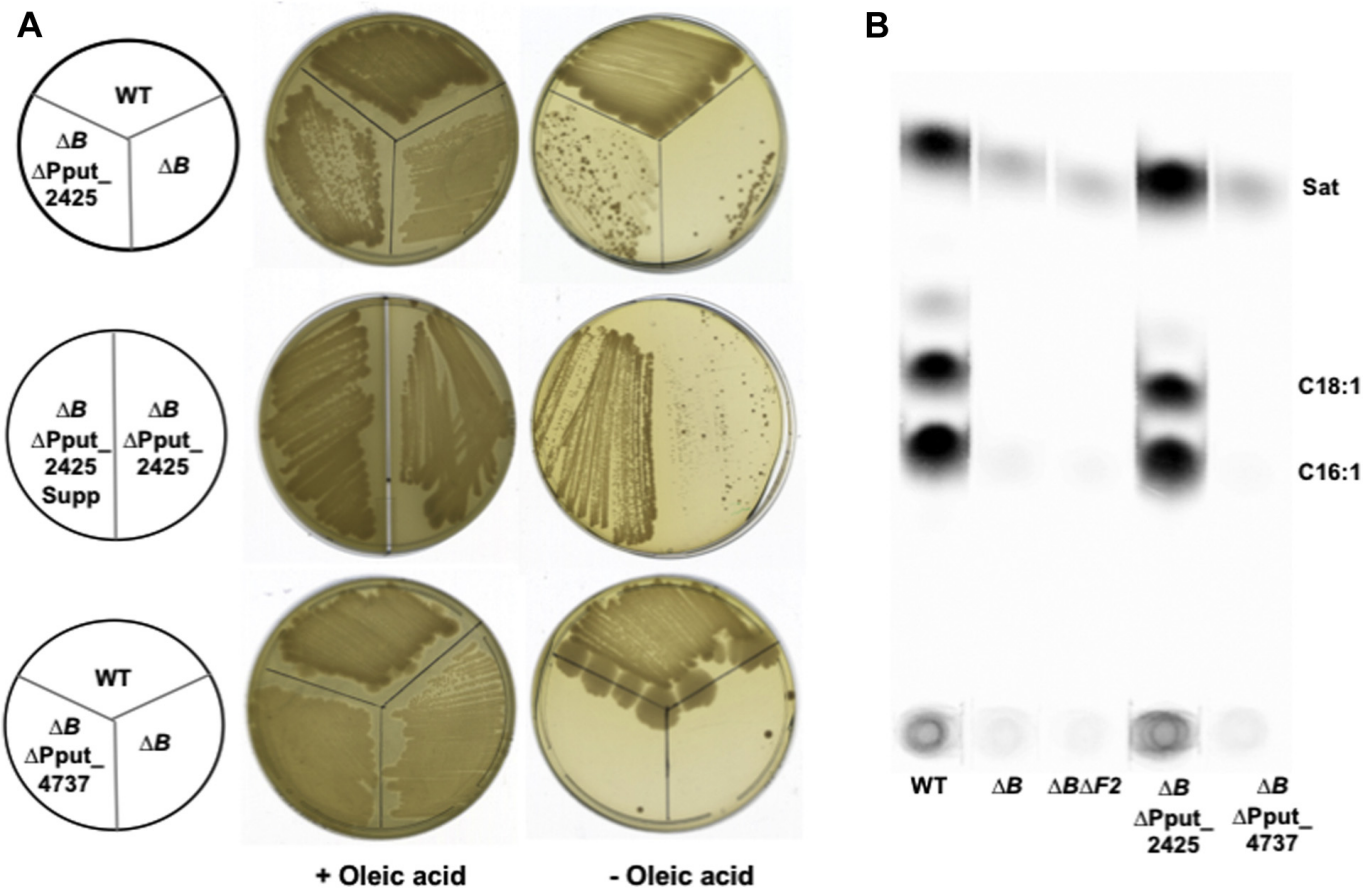
**The phenotypes of the *ΔfabB Δ*Pput_2425 and *ΔfabB Δ*Pput_4737 strains.***A*, the growth phenotype of the *ΔfabB* strain (*ΔB*), the *ΔfabB Δ*Pput_2425 strain (*ΔBΔ*Pput_2425), and the *ΔfabB Δ*Pput_4737 (*ΔBΔ*Pput_4737) strain on LB plates in the presence or absence of oleic acid. The *left side* of the *middle* plate contains ΔPput_2425Δ *fabB* suppressors (denoted as Supp), whereas the *right side* is the parental ΔPput_2425 Δ*fabB* strain. *B*, the phospholipid fatty acids of *Pseudomonas putida* F1 WT, *ΔfabB*, *ΔfabB ΔfabF2*, *ΔfabB Δ*Pput_2425, and *ΔfabB Δ*Pput_4737 strains labeled with [1-^14^C]acetate. The two mutations of the *ΔfabB Δ*Pput_2425 strain restored UFA synthesis. Panel *B* is a scan of the autoradiogram of the thin layer plate used to separate the radioactive methyl ester species. UFA, unsaturated fatty acid.

Suppression of the Δ*fabB* mutation is due to increased *fabF2* expression resulting from inactivation of Pput_2425 (Fig. 6). Another putative regulatory gene is Pput_4737, which encodes a protein that is 72.6% identical to *P. aeruginosa* PAO1 DesT, a negative regulator of UFA synthesis (24). To test the possible involvement of this regulatory protein in Δ*fabB* suppression, ΔPput_4737 and ΔPput_4737 Δ*fabB* strains were constructed. The ΔPput_4737 mutation failed to alter the phenotype of the Δ*fabB* strain. The ΔPput_4737 Δ*fabB* strain grew stably in LB medium containing 5 mM oleic acid and grew weakly with suppressor accumulation in the absence of oleic acid (Fig. 7*A*). Moreover, the ΔPput_4737 mutation failed to affect the phenotype of the Δ*fabB* strain (Fig. 7*A*). Labeling with [1-^14^C]acetate demonstrated that only in the Δ*fabB* ΔPput_2425 strain was UFA synthesis restored (Fig. 7*B*).

An interesting phenotype of the *ΔfabB* suppressor strains is that they are variably sensitive to octanoic acid (Fig. S7). A concentration of 5 mM octanoate completely inhibits growth of some suppressor strains but only partially inhibits growth of others, whereas the WT strain grows normally (Fig. S7). A possible scenario to explain growth inhibition is discussed below.

### Expression of the Pput_2425, Pput_2424, fabF2, and fabA genes in *P. putida* F1

**Figure 8:**
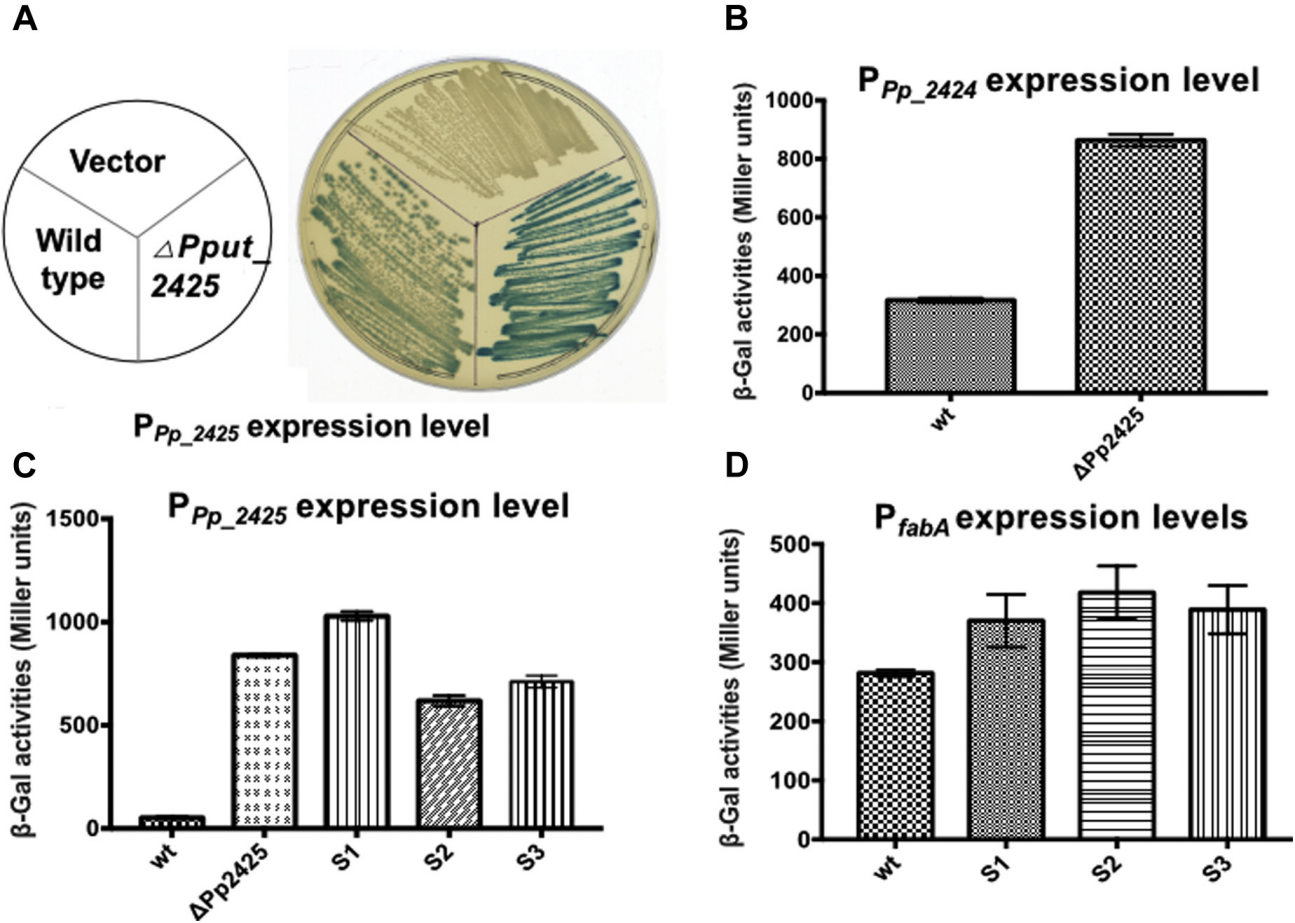
**The expression levels of P**_**Pput_2425**_**, P**_**Pput_2424**_**, and P**_***fabA***_**.***A*, the expression level of P_Pput_2425_ on LB plates containing X-gal. *B*, *β*-galactosidase activity of P_Pput_2424_ in WT and *Δ*Pput_2425. *C*, *β*-galactosidase activity of P_Pput_2425_ in WT, *Δ*Pput_2425, and three *ΔfabB* suppressor strains (S1–S3). *D*, *β*-galactosidase activity of P_*fabA*_ in WT and the three *ΔfabB* suppressor strains. X-Gal, 5-bromo-4-chloro-3-indolyl-β-D-galactoside.

The upstream 200-bp regions of Pput_2425 and *fabF2* were fused to a promoter-less *lacZ* of the pSRK-*lacZ* expression vector (25), to allow detection of possible promoters. The P_Pput_2425_ fusion plasmids were transferred into the WT strain, the ΔPput_2425 strain plus the Δ*fabB* suppressor strains, and *β*-galactosidase activity was measured. The *β*-galactosidase activity of the Pput_2425 mutant and suppressor strains was much higher than that of the WT strain (Fig. 8, *A* and *C*). The P_Pput_2424_ and P_*fabF2*_ *lacZ* fusion plasmids were then transformed into the WT and ΔPput_2425 strains. However, for P_*fabF2*_, the *β*-galactosidase activity of the ΔPput_2425 strain was only slightly higher than that of the WT strain (Fig. S8*A*), indicating that the *fabF2* upstream 200-bp segment lacked a promoter, whereas the *β*-galactosidase activity of P_Pput_2424_ was greatly increased in the ΔPput_2425 strain (Fig. 8*B*). These results indicated that Pput_2425 controls expression of itself, the *fabF2* gene cluster, and thus *fabF2* expression. Reverse transcription PCR confirmed that *fabF2* is cotranscribed with Pput_2425 (Fig. S9).

Although *fabF2* is involved in the synthesis of UFA, the ΔPput_2425 Δ*fabB* double mutant grew weakly and suppressors appeared on plates lacking oleic acid. Sequencing the *fabF1*, *fabF2*, and *fabA* genes of the suppressors plus their promoter regions gave only WT sequences. The fatty acid composition of a Δ*fabB* suppressor strain was similar to that of the WT strain although the UFA content was somewhat decreased (Table S5). The anaerobic FabA-FabB pathway is the major pathway of bacterial UFA synthesis (26). Increased expression of FabF2 improved growth and replaced *fabB* function and it seemed possible that increased expression of *fabA* might also aid UFA synthesis in the Δ*fabB* suppressor strains. The transcription start site of the *fabA* was identified by rapid amplification of 5' complementary DNA ends and was located 120 bp upstream of the *fabA* coding sequence (Fig. S10).

The upstream 200 bp of the *fabA* promoter was fused to the promoter-less *lacZ* of the expression vector. The fusion vector was transferred into WT, ΔPput_2425, and *ΔfabB* suppressor strains, and β-galactosidase activities were determined (Fig. 8*C*). The β-galactosidase activity in the *ΔfabB* suppressor strains (Fig. 8*C*) and the ΔPput_2425 strain was much higher than that of the WT strain (Fig. 8*C*). This showed that the accumulation of suppressors might also be related to the *fabA* expression level. Note that deletion of Pput_4737 had only a very modest effect on *fabA* expression (Fig. S8*B*).

Deletion of *P. aeruginosa* PAO1 *fabF1* has been reported to affect swimming mobility (27). Hence, the swimming motility phenotypes of the *P. putida* F1 Δ*fabF1* and *ΔfabF2* strains were tested. The Δ*fabF1* strain had significantly decreased swimming motility, whereas the Δ*fabF1 ΔfabF2* strain was basically immobile, indicating that *fabF1* affected swimming, whereas the Δ*fabF2* mutation aggravated the effects of *fabF1* on swimming (Fig. S11*A*). Because the genes downstream of *fabF2* are related to drug transport, we tested the resistance of the *fabF2* mutant strain to three antibiotics and found that after the Δ*fabF2* mutation, the resistance of *P. putida* F1 to ampicillin, erythromycin, and carbenicillin increased (Fig. S11*B*)

## Discussion

**Figure 9:**
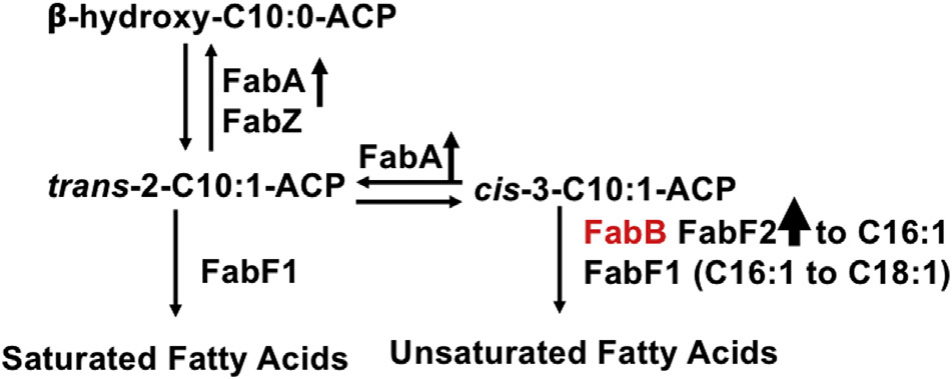
**Scheme of the *Pseudomonas putida* F1 UFA synthesis pathway.** Upon inactivation of *fabB*, greatly increased expression (*large arrow*) of *fabF2* resulting from inactivation of the Pput_2425 repressor replaced FabB function. Expression of *fabA* also was modestly increased (*small arrow*). FabB and FabF2 are unable to elongate palmitoleyl-ACP (C16;1) to *cis*-vaccenoyl-ACP (C18;1), whereas FabF1 elongates palmitoleyl-ACP (C16;1) to *cis*-vaccenoyl-ACP (C18;1) but is unable to perform the first elongation in UFA synthesis (*cis*-3-decenoyl-ACP to 3-keto, *cis*-5-dodecenoyl-ACP). ACP, acyl carrier protein; UFA, unsaturated fatty acid.

*P. putida* is a soil and water bacterium that can utilize a very wide variety of organic compounds as carbon and energy sources, and thus, *P. putida* strains are often utilized in bioremediation (1, 5). This lifestyle may provide a rationale for the complexity of UFA synthesis and regulation in *P. putida* F1 (Fig. 9) and other pseudomonads compared with the paradigm *E. coli* pathway. Degradation of a number of the utilized compounds proceeds through acyl-CoA intermediates, which could enter the fatty acid synthesis pathway and alter the UFA:SFA ratio. Intermediates in β-oxidation have been shown to enter the fatty acid synthesis pathway of *P. aeruginosa via* a 3-ketoacyl-ACP synthase encoded by the PA3286 ORF (28). *P. putida* F1 encodes a protein, Pput_1345, with 74% identity to Pa3286 and like Pa3286 elongates octanoyl-CoA with malonyl-ACP *in vitro* (data not shown). Hence, a possible scenario is that the complexity of *P. putida* UFA synthesis and regulation may be a means to cope with the diversity of acyl-CoA intermediates generated in consumption of diverse organic compounds.

The fatty acid components of *P. putida* are all straight-chain fatty acids as found in *E. coli*. At present, there are two routes for UFA synthesis in bacteria, anaerobic and aerobic pathways (although the anaerobic pathway also functions aerobically). Thus far, the anaerobic FabA-FabB is the major pathway, whereas aerobic desaturation is in general a supplementary pathway (29). Although the aerobic desaturation pathway of *P. aeruginosa* PAO1 can partially compensate for loss of the anaerobic pathway (30), this is not the case in *P. putida* F1. The *P. putida* Δ*fabB* strain is a UFA auxotroph, and hence, the aerobic pathway is unable to support growth. Indeed, deletion of the *desA* gene had little or no effect on UFA synthesis, although DesA may account for the traces of UFA synthesis seen in the *ΔfabB ΔfabF2* strain.

The Δ*fabB* strain spontaneously accumulates suppressors in which *fabF2* restores UFA synthesis. Suppressors accumulate because of inactivating mutations in a regulatory protein encoded upstream in the *fabF2* gene cluster, which determines whether the otherwise cryptic *fabF2* gene has gained enough expression to significantly participate in UFA synthesis. This regulatory protein, Pput_2425, belongs to the TetR family of transcriptional repressors, which are often involved in the regulation of fatty acid synthesis (22, 23). The *P. putida* mechanism is different from that of the Δ*fabB* suppressors of *Shewanella*. In *Shewanella, fabF1* is located in the fatty acid synthesis gene cluster, and mutations that eliminate an upstream transcriptional terminator allow increased transcription of *fabF1* that compensates for loss of *fabB* (15).

Analysis of growth phenotype of suppressors found that the growth of suppressors was significantly inhibited by exogenous octanoic acid (Fig. S7). This phenotype may result because the activity of FabF2 is lower than the activities of FabB and FabF1. As noted above, *P. putida* F1 encodes a protein that condenses octanoyl-CoA and malonyl-ACP *in vitro* (data not shown). A possible scenario that could explain the octanoic acid inhibition is as follows. Upon entry of octanoic acid into the cytosol, it becomes activated to octanoyl-CoA, which can then either enter the fatty acid synthesis pathway or be degraded by β-oxidation. The branch point for the synthesis of SFA and UFA takes place after the formation of 3-hydroxydecanoyl-ACP. The *trans* double bond is introduced by FabA or FabZ, but only FabA is capable of the isomerization of *trans*-2 to *cis*-3-decenoyl-ACP (28) (Fig. S1). Addition of octanoic acid may increase the levels of *cis*-3-decenoyl-ACP but because elongation by FabF2 is weak, excess *cis*-3-decenoyl-ACP would be isomerized back to *trans*-2-decenoyl-ACP where it would enter the SFA synthetic pathway. This may lead to an imbalance in the ratio of SFA and UFA causing growth inhibition.

DesT is a regulator of UFA synthesis in *P. aeruginosa* PAO1, where it controls expression of *fabA* (24). A *P. putida* homolog of DesT is encoded by Pput_4737. Deletion of Pput_4737 resulted in modestly increased expression of the *fabA fabB* operon as seen in *P. aeruginosa* PAO1. Upon loss of *P. putida* F1 FabB activity, there is a strong selection for inactivation of the Pput_2425 regulatory protein, which results in increased expression of *fabF2* to bypass loss of *fabB*. From the properties of *P. aeruginosa* DesT, we expected that *fabA* expression would be altered by the DesT homologous gene Pput_4737 (Fig. S8*B*). However, sequencing the upstream 200-bp regions of DesT homologous gene Pput_4737 and the *ΔfabB* suppressor strains showed no differences from the WT sequences. Although the expression of *fabA* slightly increased upon deletion of Pput_4737 (Fig. S8*B*), the properties of the ΔPput_4737 Δ*fabB* and Δ*fabB* strains were the same, indicating that Pput_4737 was not involved in suppressor accumulation. Note that, although the ΔPput_2425 mutation restored UFA synthesis of the Δ*fabB* strain, suppressors still accumulated. This may be related to *fabA* expression, but the mechanism will require further study.

Phylogenetic tree analysis showed that the homologs of *P. putida fabF2* not only exist in *Pseudomonas*, but highly homologous genes are found in other species. These genes are often located in gene clusters resembling that of *fabF2* and all such clusters contain a regulatory factor of the TetR family (Fig. S12). Perhaps, these bacteria may also compensate for the defects in UFA synthesis by activating *fabF2* expression.

## Experimental procedures

### Bacterial strains, plasmids, and growth conditions

The strains and plasmids are given in Table S1. *E. coli* and *P. putida* F1 strains were grown at 37 °C and 30 °C in LB medium containing (in g/l) tryptone, 10; yeast extract, 5; NaCl, 10; pH 7.0. When required, antibiotics and inducers were added as follows (in μg/ml): sodium ampicillin, 100; kanamycin sulfate, 30; gentamicin, 30; tetracycline hydrochloride, 90; L-arabinose, 200; IPTG, 240; and 5-bromo-4-chloro-3-indolyl-β-D-galactoside (X-Gal), 20. Oleate was used at a final concentration of 5 mM. Bacterial growth was determined by growing on solid media.

### Expression and purification of His_6_-tagged FabB, FabF1, and FabF2 proteins

Vector pET28b constructs carrying with *fabF1*, *fabF2*, and *fabB* were transformed into strain BL21(DE3). The transformants were incubated in LB medium at 37 °C with 50 μg/ml kanamycin to an absorbance at 600 nm of 0.6 and then were induced by 1 mM IPTG for another 4-h incubation. The cells were harvested and lysed in the lysis buffer (50 mM sodium phosphate (pH 8.0), 300 mM NaCl, and 10 mM imidazole). The supernatant was loaded onto the Ni-NTA column. The column was eluted with a wash buffer (50 mM sodium phosphate (pH 8.0), 300 mM NaCl, and 40 mM imidazole), and then the tagged proteins were eluted with the same buffer containing 250 mM imidazole. The eluted proteins were dialyzed against 50 mM sodium phosphate (pH 8.0) and 300 mM NaCl, glycerol was added after dialysis to 15%, and the proteins were stored at −80 °C.

### Assay of long-chain 3-ketoacyl-ACP synthase activities *in vitro*

The PCR products containing *E. coli acpP* and *acpS* were cloned into pET-28b or pBAD33 to yield plasmids pET-ACP and pBAD33-AcpS, respectively. These two plasmids were introduced into *E. coli* BL21(DE3) cells, and holo-ACP was expressed at high levels and purified. The abilities of FabF2 and FabB to function in the cycle of fatty acid synthesis were assessed with reaction mixtures containing 0.1 M sodium phosphate (pH 8.0); 0.1 μg each of EcFabD, EcFabG, EcFabA, and EcFabI; 50 μM NADH; 50 μM NADPH; 1 mM β-mercaptoethanol; 100 μM acyl-ACP; 100 μM malonyl-CoA; and 50 μM holo-ACP in a final volume of 40 μl. The reactions were initiated by the addition of FabF2 or FabB to the mixture, followed by incubation for 1 h. The reaction products were resolved by conformationally sensitive gel electrophoresis on 13.5% polyacrylamide gels containing a concentration of urea optimized for separation.

### Thin layer chromatography analysis of phospholipid fatty acids

The complemented K1060 derivatives and the *P. putida* F1 strains and their complemented derivatives were cultured in LB medium with or without oleic acid and labeled with radioactive [1-^14^C]acetate as follows. The strains were grown to an absorbance at 600 nm 0.5 with 5 mM oleic acid, and the cells were washed with 0.5% Brij 58 three times to remove oleate, resuspended in media lacing oleate, and incubated for another 3 h at 37 °C (K1060) or 30 °C (*P*. *putida* F1) in the presence of [1-^14^C]acetate (final concentration of 1 μCi/ml) followed by cell lysis with methanol–chloroform (2:1). The phospholipids were further extracted with chloroform and dried under nitrogen. The fatty acyl groups on phospholipids were then converted to their methyl esters by transesterification with 25% sodium methoxide, extracted into petroleum ether, taken to dryness under nitrogen, resuspended in hexanes, and loaded onto the silver nitrate thin layer chromatography plates (Analtech), which were developed in toluene at −20 °C (inclusion of silver allows separation of saturated and unsaturated esters with different double bond positions). The plates containing the [1-^14^C]-labeled esters were analyzed by phosphorimaging using a GE Typhoon FLA 7000 Scanner and analyzed by the ImageQuant TL program.

### GC-MS analysis of phospholipid fatty acids

The strains were cultured in LB medium to an absorbance at 600 nm 0.5. Cultures were standardized by absorbance at 600 nm, and fatty acid methyl esters were generated as above and then analyzed by GC-MS using a highly polar chiral CP-Si 88 column (Agilent Technologies). The CP-Si88 column allows baseline separation of the methyl esters of acids based on their double-bond positions.

### Extraction of total RNA, cDNA synthesis, and reverse transcription PCR

Total RNA was purified using the RNeasy Mini Kit (Qiagen). RNAs were nonspecifically converted to single-stranded cDNAs using the ProtoScript First Strand cDNA Synthesis Kit (NEB). The control samples were made during cDNA synthesis from the total RNA using the ProtoScript First Strand cDNA Synthesis Kit without the addition of reverse transcriptase. The resulting cDNA served as the template for PCR amplification of the Pput_2425 gene cluster, using specific primers (Table S2) and an Eppendorf thermal cycler.

### RLM-RACE

The 5’ends of *fabA* mRNA in *P. putida* F1 were mapped using RLM-RACE using the First-Choice RLM-RACE kit (ThermoFisher) according to the manufacturer’s instructions. To identify the 5′ends of the *fabA* mRNA, the PCR products were cloned into vector PCR 2.1 and sequenced.

### Swimming and antibiotic resistance assays

The swimming assays were performed on a semi-solid plate containing 0.3% agarose, and the plate was allowed to air-dry on a clean bench for 5 to 10 min before use. The bacteria were first cultured in liquid LB medium to the stationary phase and transferred to 5-ml fresh liquid LB medium at a ratio of 1:100 to allow the culture to achieve the log phase, and the absorbance at 600 nm value was determined. The test culture was diluted to an absorbance at 600 nm 0.5, and 1 μl of the bacteria solution for each sample was placed on the swimming plate or plates containing the antibiotics to be tested. Growth of the lawn was observed after placing them at 25 °C for 12 h to 16 h. Three repetitions were performed.

## Data availability

All data are contained within the article.

## Supporting information

This article contains supporting information.

## Conflict of interest

The authors declare that they have no conflicts of interest with the contents of this article.
