# Supplementary material for: A cryptic long-chain 3-ketoacyl-ACP synthase in the Pseudomonas putida F1 unsaturated fatty acid synthesis pathway
Source: J Biol Chem. 2021 Jun 25;297(2):100920. doi: 10.1016/j.jbc.2021.100920 (PMC8319022; doi:10.1016/j.jbc.2021.100920)
Supplement: Supplemental Figures S1–S12 and Tables S1–S5 [file mmc1.pdf]

Supplement to:

A Cryptic Long-Chain 3-Ketoacyl-ACP Synthase in the Unsaturated Fatty Acid Synthetic Pathway of *Pseudomonas putida* F1

Huijuan Dong, Jincheng M, Qunyi Chen, Bo Chen, Lujie Liang, Yuling Liao Yulu Song[1]
Haihong Wang[1*], John E. Cronan[2,3*]

Materials and Methods

Construction of *P. putida* F1 *fabF1, fabF2 and fabB* deletion strains

PCR amplification was performed to obtain DNA fragments of about 500 bp both upstream and downstream of *fabF1* or *fabF2*. The DNA fragments were fused by overlap extension PCR. These DNA fragments were cloned into the suicide vector pK18mobsacB carrying Km resistance to form a suicide plasmid. After confirmation by DNA sequencing the recombinant plasmid was transformed into *E. coli* S17-1 strain to obtain a donor strain for conjugation. The donor bacteria S17-1 (37℃) and the receptor *P. putida* F1 (30℃) were separately cultured in 5 ml liquid LB medium, respectively, and the cells were collected by centrifugation, respectively. The bacteria were washed in fresh LB medium 3 times, then suspend each cell pellet in 0.1 ml LB medium, mix the bacterial suspension on a LB solid medium plate and culture at 30℃ for 24~48 h. The bacterial suspension was collected, diluted appropriately, and spread on LB solid plates with Km (30 μg/mL) and Amp (100 μg/mL) and incubated at 30℃ for 24~48 h. A single recombinant colony was selected and cultured in LB liquid medium for 24~48 h. The culture was appropriately diluted and applied to LB solid medium (containing 10% sucrose). Single colonies from the LB-sucroseplates were screened for the expected gene mutations and sequences with the wild type strains used as controls.

The construction of the *fabB* mutant proceed identically except that a Km resistance cassette was ligated between the upstream and downstream *fabB* sequences to improve screening efficiency. The pKD4 plasmid was used as a template to obtain a fragment containing a FRT locus and a Km resistance cassette, and the plasmid fragment was amplified by PCR using the pK18mobsacB plasmid carrying Gm resistance as the template. PCR was performed to amplify

about 500 bp of the sequences upstream and downstream of the *fabB* gene. Fusion of the multiple fragments was performed using the NEB Gibson Assembly Kit to obtain the *fabB* knockout vector carrying Km resistance. The Δ*fabB* strain was obtained by screening on LB-sucrose plates containing Km and 5 mM oleic acid then verified by PCR and sequencing.

Construction of complementation strains

The targeted gene fragment was amplified by PCR or overlapping PCR, digested with *Nde*I and *Hin*dII, and ligated into the pSRK vector digested with the same enzyme. After screening ann verification the plasmids were transferred to the host cell by conjugation (as above) or electroporation. Electroporation was performed as follows: a 5 mL culture of the host strain was grown overnight, 1 mL of the host strain culture was washed twice with 300 mM sucrose, concentrated to 100 μL, the complementation plasmid was added. The solution was transfered to the electroporation cuvette and put on ice for 20 min, after a 2.5 KV shock, 1 mL fresh medium was added and after culturing for 1 h at 30℃ plated in media containing the appropriate selective antibiotic.

ß-Galactosidase assays

To construct the *lacZ* translational fusions in *P. putida* F1 the 5' fragments promoter of P_{Pput_2425} were fused in the correct translational reading frame to a large 3' fragment of *lacZ* gene in vector pSRK Tc. The P_{Pput_2425} fusion vector was transferred to the wild type and the Δ *fabB* suppressor strains and the blue colonies were detected on the 0.1 mg/ml X-gal containing plates. The 5' fragments promoter of P_{fabA}, P_{Pput_2424} and P_{fabF2} were also fused with pSRK Tc, then they were transferred to wild type strain, ΔPput_4737 or ΔPput_2425 strains. ß-Galactosidase assays were also performed as described by Miller. Mid- log-phase cultures were collected by centrifugation, washed twice with Z buffer, and assayed for ß-galactosidase activity after lysis with sodium dodecyl sulfate-chloroform. The data were obtained in triplicate in more than three independent experiments.

Table S1. Strains and Plasmids

***E. coli* Strains**		
DH5α	φ80d lacZΔM15 endA1recA1hsdR17(rK-, mK+)	Lab stock
S17-1	Tp^r Sm^r *recA*, *thi*, *pro*, *hsdR*$^-$*M*$^+$ RP4::2-Tc::Mu:Km ::Tn*7*, *λpir*	Lab stock
CY242	*fabB*(Ts)	Lab stock
CY244	*fabB*(Ts)*fabF*	Lab stock
K1060	*fabB5*	Lab stock
CL28	*fabF* :: Kan	Lab stock
BL21(DE3)	*omp*T *hsd*S B (rB$^-$ mB$^-$)	Lab stock
***P. putida* Strains**		
F1	wild type	Lab stock
HJ30	Δ*fabF1*	This work
HJ27	Δ*fabF2*	This work
HJ28	Δ*fabF3*	This work
HJ29	Δ*fabF4*	This work
HJ102	Δ*fabF1* Δ*fabF2*	This work
HJ289	Δ*fabF1* Δ*fabF 2*Δ*desA*	This work
HJ341	Δ*fabF1* Δ*fabF2*Δ*desA* (*fabF2* replaced *fabB)*	This work
HJ211	Δ*fabB*	This work
HJ302	Δ*fabB* Δ*fabF2*	This work
HJ34	Δ*fadD1* Δ*fadD2*	This work
HJ385	Δ*fadD1* Δ*fadD2* Δ*fabB*	This work
HJ370	Δ*fadB*Δ*fadA*	This work
HJ383	Δ*fadB* Δ*fadA*Δ*fabB*	This work
HJ346	ΔPput_2425	This work
HJ347	ΔPput_4737	This work
HJ396	Δ*fabB* ΔPput_2425	This work
HJ386	Δ*fabB* ΔPput_4737	This work

Plasmids		
pBAD24M	Amp^r, expression plasmid	Lab stock
pET28(b)	Kan^r, expression plasmid	Lab stock
pK18 mobsacB A	Kan^r, suicide plasmid	Lab stock
pK18 mobsacB B	Gm^r, suicide plasmid	Lab stock
pSRK	Gm^r, expression plasmid	Lab stock
pSRK-*lacZ*	Tc^r, P*cti-lacZ* expression plasmid	Lab stock
pHJ1	*P. putida* F1 *fabB* expression plasmid derived from pBAD24M	This work
pHJ2	*P. putida* F1 *fabF1* expression plasmid derived from pBAD24M	This work
pHJ3	*P. putida* F1 *fabF2* expression plasmid derived from pBAD24M	This work
pHJ4	*P. putida* F1 *fabF3* expression plasmid derived from pBAD24M	This work
pHJ5	*P. putida* F1 *fabF4* expression plasmid derived from pBAD24M	This work
pHJ6	*P. putida* F1 *fabB* expression plasmid derived from pET28(b)	This work
pHJ7	*P. putida* F1 *fabF2* expression plasmid derived from pET28(b)	This work
pHJ8	*P. putida* F1 *fabF1* knockout cassette in vector pK18mobsacB	This work
pHJ9	*P. putida* F1 *fabF2* knockout cassette in vector pK18mobsacB	This work
pHJ10	*P. putida* F1 *fabF3* knockout cassette in vector pK18mobsacB	This work
pHJ11	*P. putida* F1 *fabF4* knockout cassette in vector pK18mobsacB	This work
pHJ12	*P. putida* F1 *fabB* knockout cassette (with Kan^R resistance marker) in vector pK19mobsacB	This work

pHJ13	*P. pseudomonas* F1 *fabB* knockout cassette (with *fabF2* and Kan^R resistance marker) in vector pK19mobsacB	This work
pHJ14	*P. putida* F1 Pput_4737 knockout cassette in vector pK18mobsacB	This work
pHJ15	*P. putida* F1 *PpfadBfadA* knockout cassette in vector pK18mobsacB	This work
pHJ16	*P. putida* F1 *PpfadD1* knockout cassette in vector pK18mobsacB	This work
pHJ17	*P. putida* F1 *PpfadD2* knockout cassette in vector pK18mobsacB	This work
pHJ18	*P. putida* F1 Pput_2425 knockout cassette in vector pK18mobsacB	This work
pHJ19	*P. putida* F1 *fabF1* expression plasmid derived from pSRK	This work
pHJ20	*P. putida* F1 *fabF2* expression plasmid derived from pSRK	This work
pHJ21	*P. putida* F1 *fabF3* expression plasmid derived from pSRK	This work
pHJ22	*P. putida* F1 *fabF4* expression plasmid derived from pSRK	This work
pHJ23	*P. putida* F1 *fabB* expression plasmid derived from pSRK	This work
pHJ24	*P. putida* F1 *fabF2* promoter fused to *lacZ* in vector pSRK-*lacZ*	This work
pHJ25	*P. putida* F1 *fabA* promoter fused to *lacZ* in vector pSRK-*lacZ*	This work
pHJ26	*P. putida* F1 Pput_2425 promoter fused to *lacZ* in vector pSRK-*lacZ*	This work
pHJ27	*P. putida* F1 Pput_2424 promoter fused to *lacZ* in vector pSRK-*lacZ*	This work

Table S2. Oligonucleotide primers

Primers*	Sequence 5'-3'
*PpfabF1 Nde*I	GGAATTCCATatgcaatggcaagagactc
*PpfabF1 Hin*dIII	CCCAAGCTTtcagtcggcaaaccggcgg
*PpfabF2 Nde*I	GGAATTCCATatggctcacaacgttattc
*PpfabF2 Hin*dIII	CCCAAGCTTtcatacgctggcctcccag
*PpfabF3 Nde*I	GGAATTCCATatgaaacaggcggtcgcga
*PpfabF3 Hin*dIII	CCCAAGCTTtcagcggcagtcctgagtg
*PpfabF4 Nde*I	GGAATTCCATatgagccccacacggatcg
*PpfabF4 Hin*dIII	CCCAAGCTTtcatcgcggcgcctcccgg
*PpfabB Ned*I	CTGCATATGCGCCGCGTCGTTATCAC
*PpfabB Hin*dIII	GTAAGCTTACTTGCCTTCCCAGCGCT
*PpfabF1 Bam*HI up1	CGGGATCCATACTGCAACTCAATGTGAC
PpfabF1 dn1	AGAGTCTCTTGCCATTGCAT
PpfabF1 up2	ATGCAATGGCAAGAGACTCTAATCAATAGCCAGATG GCC
*PpfabF1 Hin*dIII dn2	CCCAAGCTTGAAACAGATTGCTGTATACC
*PpfabF2 Eco*RI up1	GGAATTCATACTGCAGGTGCGCTCTAT
PpfabF2 dn1	CACGATGCGCTTTTGAATAA

PpfabF2 up2	TTATTCAAAAGCGCATCGTGTTCGGATTCGGTGGTGTGAA
*PpfabF2 Hin*dIII dn2	CCCAAGCTTAAACGTACCCACCACGAAAA
*PpfabF3 Eco*RI up1	GGAATTCTTTTGTCGTGCTCGAAACCC
PpfabF3 dn1	ATCGCGACCGCCTGTTTCAT
PpfabF3 up2	ATGAAACAGGCGGTCGCGATTCAAGTGCTCTACAGCGCAA
*PpfabF3 Hin*dIII dn2	CCCAAGCTTGATGTCGTCGAACTGCTTTT
*PpfabF4 EcoR*I up1	GGAATTCTTTTTGCTCCCAGGGCAATG
PpfabF4 dn1	ATACCCGTGATCACGATCCG
PpfabF4 up2	CGGATCGTGATCACGGGTATGAGCAACTCCTTCGGTTTCG
*PpfabF4 Hin*dIII dn2	CCCAAGCTTACACCTTGCATATAGGCGTT
*PpfadBfadA Bam*HI up1	TCGCGGATCCtcggagaatgtgtgcacacc
PpfadBfadA dn1	Cgttgagctcgatgccgttgatggcggcaa
PpfadBfadA up2	Caacggcatcgagctcaacgaagccttcgc
*PpfadBfadA Xba*I dn2	TTGCTCTAGAgacggactcctggaacatcg
*PpfadD1 Bam*HI up1	CGGGATCCgaaggataagtacccagcc
PpfadD1 dn1	Caccatcttcttcacgtacttg
PpfadD1 up2	Agtacgtgaagaagatggtgtgcaaggtcatcgacgac
*PpfadD1 Hin*dIII dn2	CCCAAGCTTtcaggcgatcttcttcaa
*PpfadD2 Bam*HI up1	CGGGATCCgacttctggaatgacaag
PpfadD2 dn1	Catcttcttcaacttgtcgacca
PpfadD2 up2	Tcgacaagttgaagaagatggcgggtactgcgttcaag
*PpfadD2 Hin*dIII dn2	CCCAAGCTTtcacgctatatcgcgcaa
PpfabB up1 (Gibson)	atgattacgaattcgagctcggtacccggggatccGACGGCTATGAAGCCACCCGA
PpfabB dn1	cgaagttcctattctctagaaagtataggaacttcgaTCACAGGTTTTCGGTGACGGTAG
PpfabB up2	tattcatatggaccatggctaattcccatgtcagccgACACGGTCATGAGCAACAGCTT

PpfabB dn2 (Gibson)	cacgacgttgtaaaacgacggccagtgccaagcttTTTGTCGTGGAGTGTGCTGCA
pKD4 up (Gibson)	TGAtcgaagttcctatactttctag
pKD4 dn (Gibson)	Cggctgacatgggaatta
pK18 mobsacB up (Gibson)	Aagcttggcactggccgtcgtttt
Pk18 mobsacB dn (Gibson)	Ggatccccgggtaccgagct
PpfabB/fabF1 dn1 (Gibson)	GCGAATAACCCTTAGAAATTGTCAGTGGAG
PpfabB/fabF1 up2 (Gibson)	GCGTGTTCACCTCCACTGACAATTTCTAAGGGTTATTCGCatggctcacaacgttatt
PpfabB/fabF1 dn2 (Gibson)	cgaagttcctattctctagaaagtataggaacttcgaTCAtcatacgctggcctcccag
PpfabB/fabF1 up3 (Gibson)	tattcatatggaccatggctaattcccatgtcagccgGACGCTGCTGCTGTCAATGAA
Pput_4737 *Xba*I up1	cgtTCTAGAAACACCTGACGTCCGAAGCGC
Pput_4737 dn1	CATGGTAGCAGAAGTGGAGaacaaagcccggtcaatgg
Pput_4737 up2	ccattgaccgggctttgttCTCCACTTCTGCTACCATG
Pput_4737 dn2 *Hin*dIII	cgtAAGCTTGGGCGAACTGCTGGTCGATGA
Pput_2425 up1 *Xba*I	cgtTCTAGAGCTCGTAACTGCTCCCGTAGC
Pput_2425 dn1	TGTCAATGGTCACAATCTGaagcgtcctggaacgggtt

Pput_2425 up2	aacccgttccaggacgcttCAGATTGTGACCATTGACA
Pput_2425 dn2 *Hin*dIII	cgtAAGCTTCGTAAGACGATGCCGAGACCG
Pput_2422-2423 RT up	TTCGACGCTGTGGTCGAGG
Pput_2422-2423 RT dn	Acgccagtgccttcgacgatat
Pput_2423-2424 RT up	TGGCGAGCGCGCCATTAGGTG
Pput_2423-2424 RT dn	TTGCGGGCCTGGTAGAACTC
Pput_2424-2425 RT up	GAGATCCTGATACAGGGCCGC
Pput_2424-2425 RT dn	ATCAAGGGCTGAGCGCGT
PpfabA (Pput_1693) out primer	AAACCTTGCGGTCGATCAGT
PpfabA inner primer	TGTATTCCGGGTTGAAACGGATA
pSRK(*lacZ*) Tc up (Gibson)	GCGCTTGGCGTAATCATGGT
pSRK(*lacZ*) Tc dn (Gibson)	GCAATTAACCCTCACTAAAGGGAAC
$P_{PpfabF2}$ fusion *lacZ* (Gibson) up	ACCGGGTCGAATTTGCTTTTCTAGAGCTCTTGCTTACGGCATTGGCAG
$P_{PpfabF2}$ fusion *lacZ* (Gibson) dn	CACGACGTTGTAAAACGACAAGCTTGGGGTGAGCCATTTCGACCTCTCG
P_{PpfabA} fusion *lacZ* (Gibson) up	ACCGGGTCGAATTTGCTTTTCTAGAGCGAAGGGCAAGCTGAACATGGCC
P_{PpfabA} fusion *lacZ* (Gibson) dn	CACGACGTTGTAAAACGACAAGCTTGGGGCGGCGCATGCGAATAACCC

P_{Pput_2424} fusion *lacZ* (Gibson) up	ACCGGGTCGAATTTGCTTTTCTAGAGCCTGGCCATCTTTCTGATCATGAGG
P_{Pput_2424} fusion *lacZ* (Gibson) dn	CACGACGTTGTAAAACGACAAGCTTGGGCAGGCGCATGGGATCCCTTT
P_{Pput_2425} fusion *lacZ* (Gibson) up	ACCGGGTCGAATTTGCTTTTCTAGAGCGCACTACTGATGTAATGATGGG
P_{Pput_2425} fusion *lacZ* (Gibson) dn	CACGACGTTGTAAAACGACAAGCTTGGGtttagtcattggaagcgtcct

*The underline indicates the restriction enzyme cut sites.

Table S3. Fatty acid compositions of the complemented Δ*fabF1*, Δ*fabF2* and Δ*fabF1* Δ*fabF2* strains at 30℃

Fatty acids %	Wild type	*ΔfabF1* /p*fabF1*	*ΔfabF1fabF2* /p*fabB*	*ΔfabF1fabF2* /p*fabF1*	*ΔfabF1fabF2* /p*fabF2*	*ΔfabF1fabF2* /p*fabF3*	*ΔfabF1fabF2* /p*fabF4*
$C_{16:0}$	17.1±7.0	15.8±2.6	20.6±0.8	13.5±1.4	18.7±1.1	18.0±5.6	18.2±2.5
$C_{16:1}$	52.3±4.1	25.2±0.8	75.3±0.6	26.8±0.9	76.4±1.9	75.8±5.3	76.7±2.3
$C_{18:0}$	0.4±0.3	1.1±0	0.5±0.1	1.0±0.1	0.9±0	1.4±0.4	1.6±0.1
$C_{18:1}$	30.2±3.4	57.9±2.1	3.6±0.1	58.7±1.5	4.5±0.2	4.7±0.9	4.6±0.5

The wild type strain carried the empty vector plasmid

Table S4. Fatty acid compositions of the strain in which *fabF2* replaced *fabB*.

Fatty acids %	Wild Type	*ΔF1 ΔF2 ΔdesA*	*ΔF1 ΔF2 ΔdesA* *fabF2* replaced *fabB*
$C_{16:0}$	14.4±0.5	25.6±5.6	29.7±3.7
$C_{16:1}$	41.8±2.0	69.4±6.0	63.4±3.1
$C_{18:0}$	-	0.7±0.2	-
$C_{18:1}$	43.8±1.5	4.4±0.3	6.4±0.4

ΔF1 ΔF2 denotes that both FabF1 and FabF2 were deleted.

Table S5. Comparison of the fatty acid compositions of the wild type strain and a Δ*fabB* suppressor strain at 30℃.

Fatty acids %	Wild Type	Suppressor
$C_{16:0}$	20.8±3.0	30.5±1.7
$C_{16:1}$	49.4±4.1	45.2±0.4
$C_{18:0}$	0.5±0.2	0.5±0.5
$C_{18:1}$	29.2±1.6	23.7±1.3

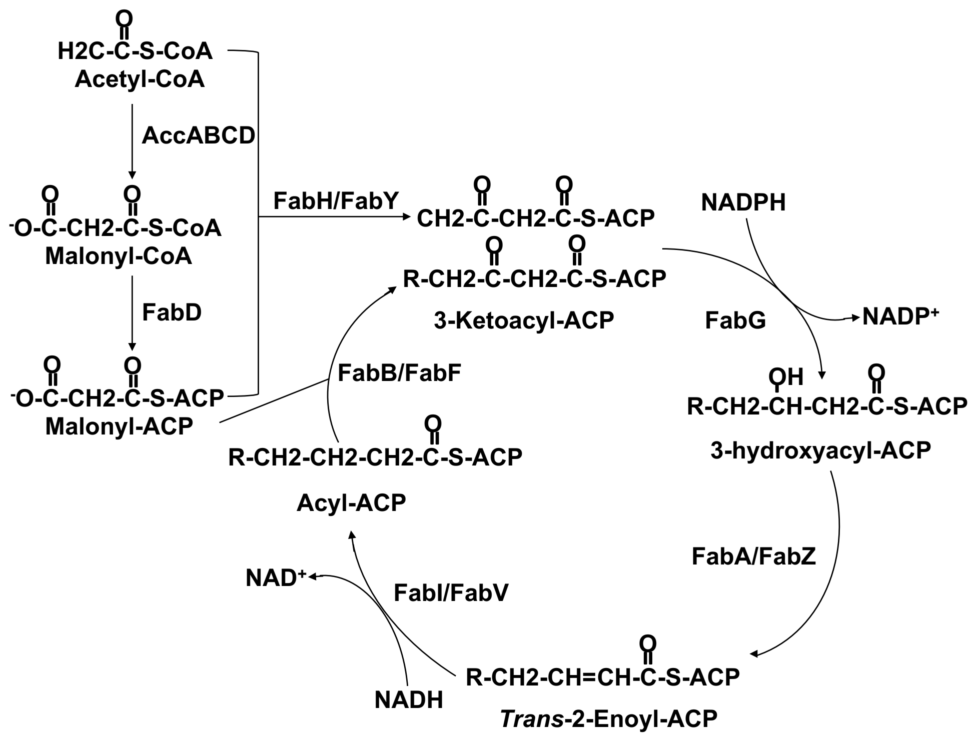

Figure S1. The fatty acid synthesis pathway in *Pseudomonas*. Abbreviations: AccABCD, acetyl-CoA carboxylase; FabD, malonyl-CoA:ACP transacylase; FabH and FabY, 3-ketoacyl ACP synthase III; FabG, 3-ketoacyl-ACP reductase; FabZ, 3-hydroxyacyl-ACP dehydratase; FabA, 3-hydroxydecanoyl-ACP dehydratase/isomerase; FabF, 3-ketoacyl-ACP synthase II; FabB, 3-ketoacyl-ACP synthase I; FabI and FabV, enoyl-ACP reductase.

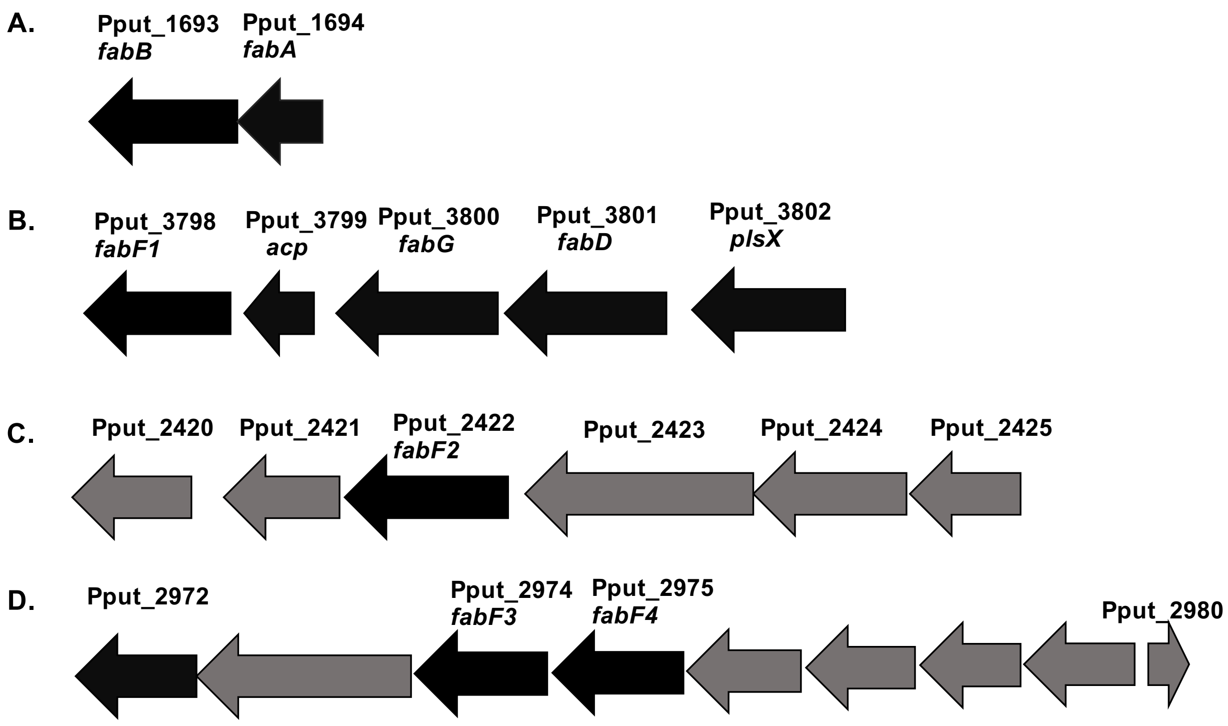

Figure S2. **The genome locations of the five candidate long-chain 3-ketoacyl ACP synthase genes of *P. putida* F1**

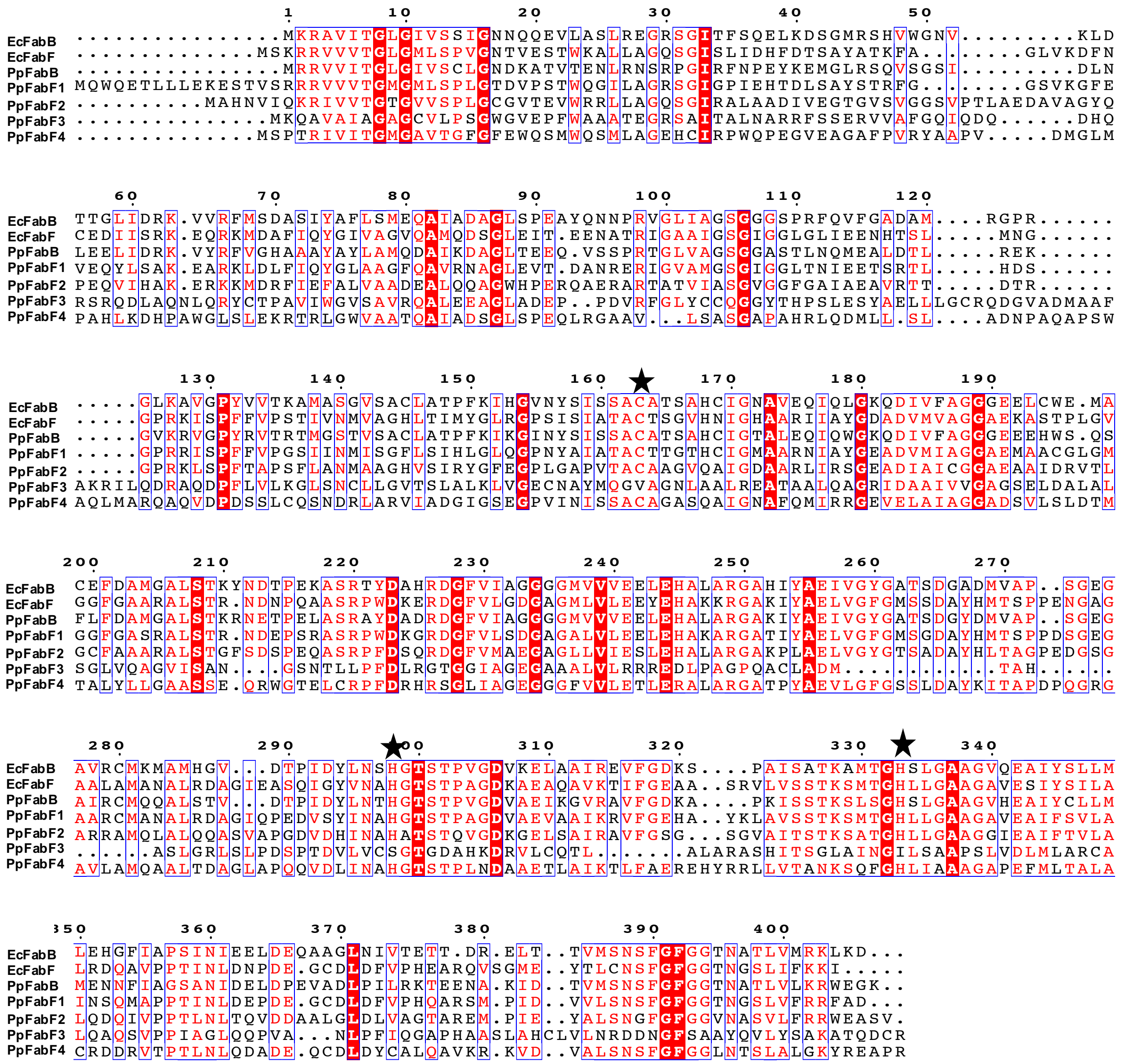

Figure S3. Alignments of PpFabB and PpFabFs with *E. coli* FabB and FabF.

Strictly conserved amino acid residues in both FabB and FabF proteins are in dark red whereas highly conserved residues are in light red. The catalytic residues conserved in KAS I and II proteins are highlighted with black stars.

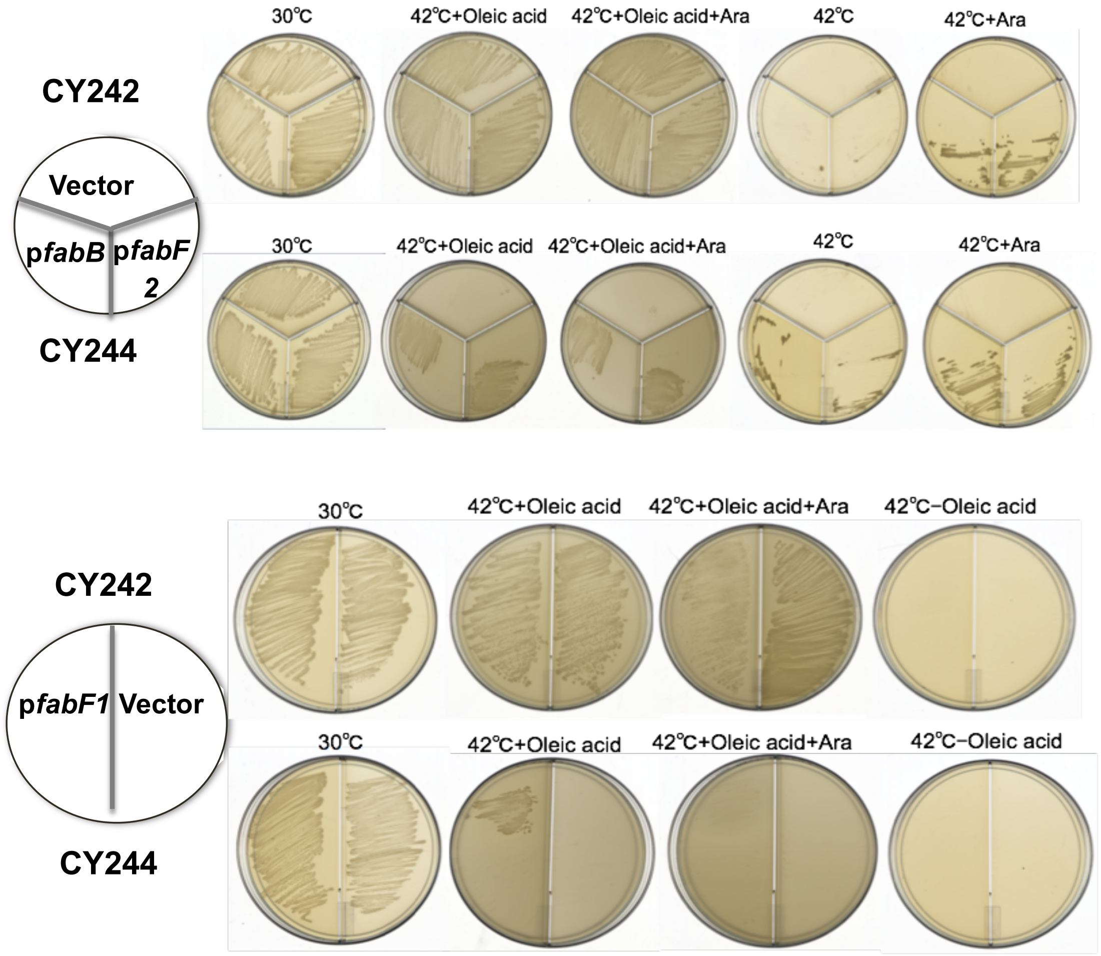

Figure S4. Complementation of *E. coli fabB* (Ts) and *fabF* mutant strains CY244 and CY242 by expression of *P. putida* F1 *fabB* and *fabF* proteins. *P. putida* F1 *fabB* and *fabF2* both complemented CY242 and CY244 growth at the non-permissible temperature; *P. putida* F1 *fabF1 required* oleic acid to complement CY244 growth at non-permissible. Overexpression of *fabF1* inhibited CY244 and CY242 growth; Ara, 0.02% arabinose.

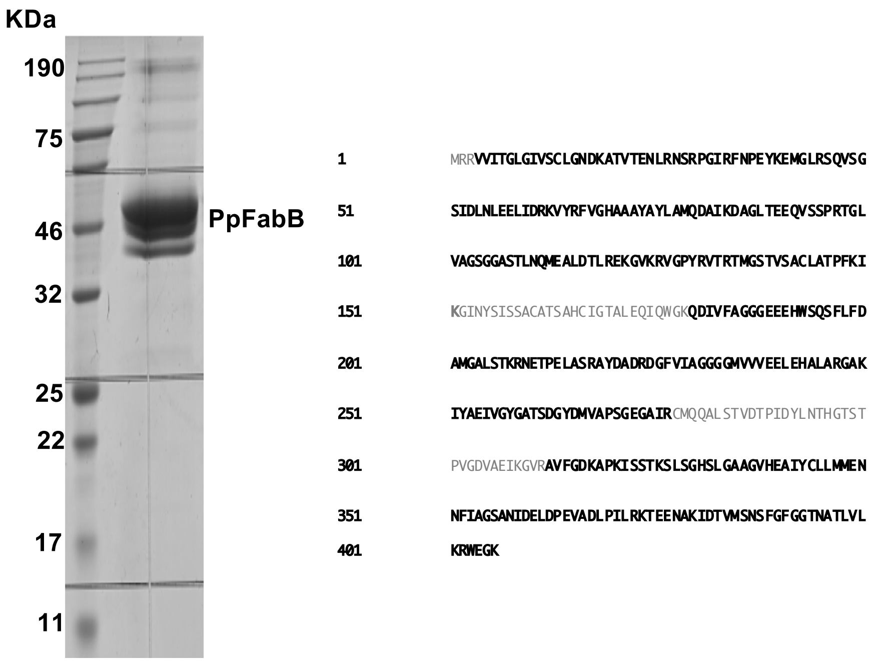

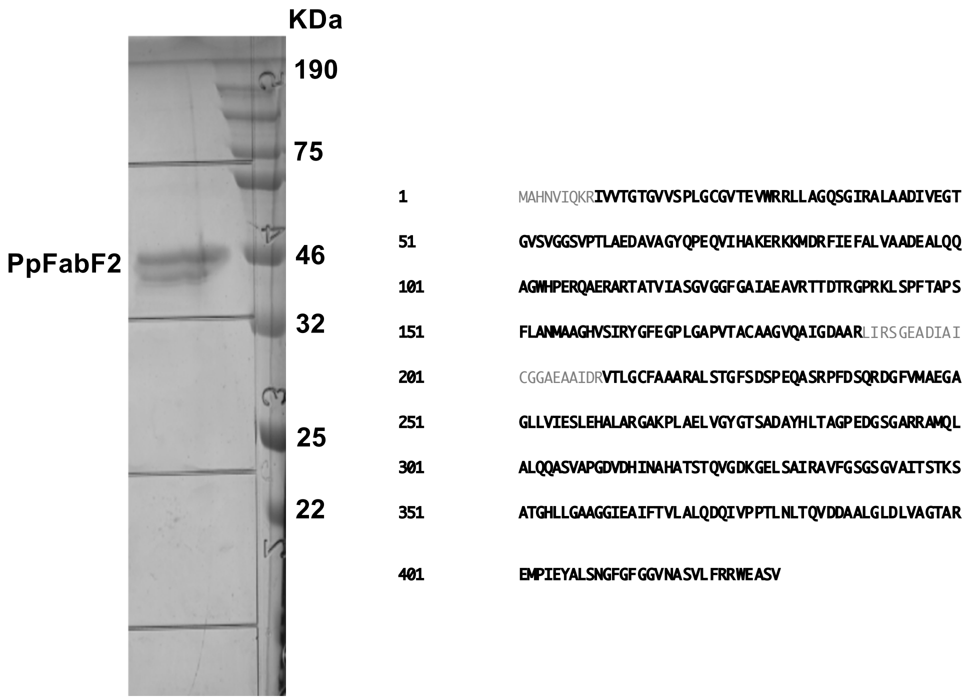

Fig. S5. Purification of *P. putida* FabB and FabF2.

Sodium dodecyl sulfate polyacrylamide gels of the purified proteins together with the tryptic peptides of each protein as determined by HPLC-MS done as previously described (32}. The recovery of peptides was excellent, 83% for FabF1 and 93% for FabF2.

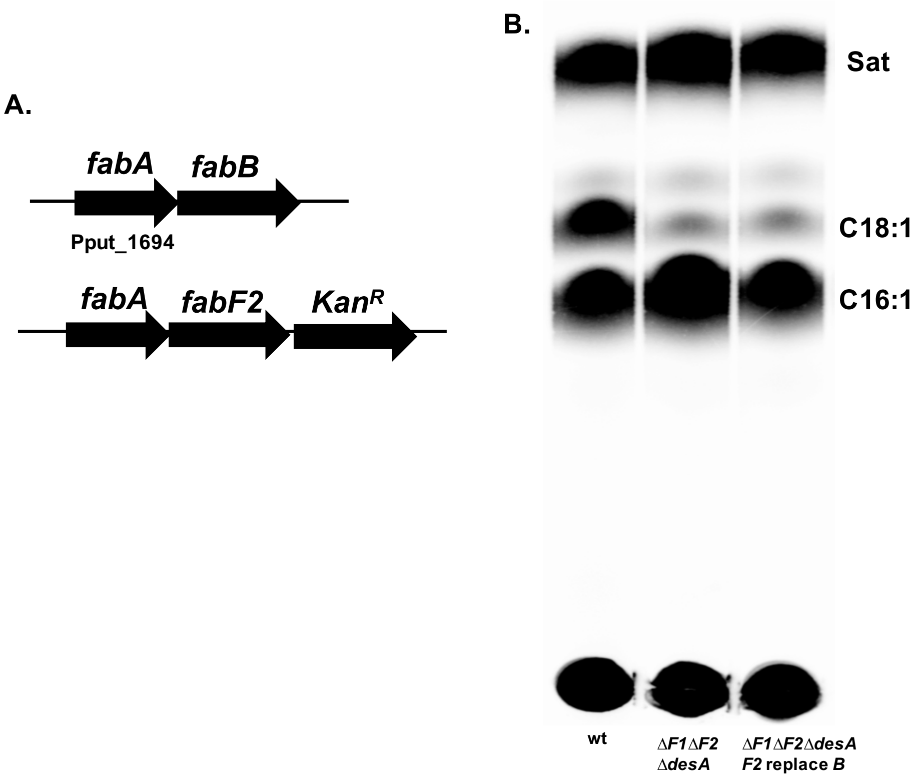

Figure S6. ***P. putida*** **F1** ***fabF2*** **replaces** ***fabB*** **function in the** **Δ*fabF1* Δ*fabF2* Δ*desA*** **triple mutant strain**. **A**. Schematic diagram of the construction of *fabF2* and *kan*^{R} replace of *fabB* in Δ*fabF1* Δ*fabF2* Δ*desA* (Δ*F1F2desA*); **B.** The phospholipid fatty acids of the wild type, Δ*fabF1* Δ*fabF2* Δ*desA* (Δ*F1F2des*) and Δ*fabF1* Δ*fabF2* Δ*desA fabF2* replace *fabB* (Δ*F1* Δ*F2* Δ*desA* (*F2* replace *B*) The fatty acids of the Δ*fabF1* Δ*fabF2* Δ*desA fabF2* strains labeled with [1-^{14}C]acetate.

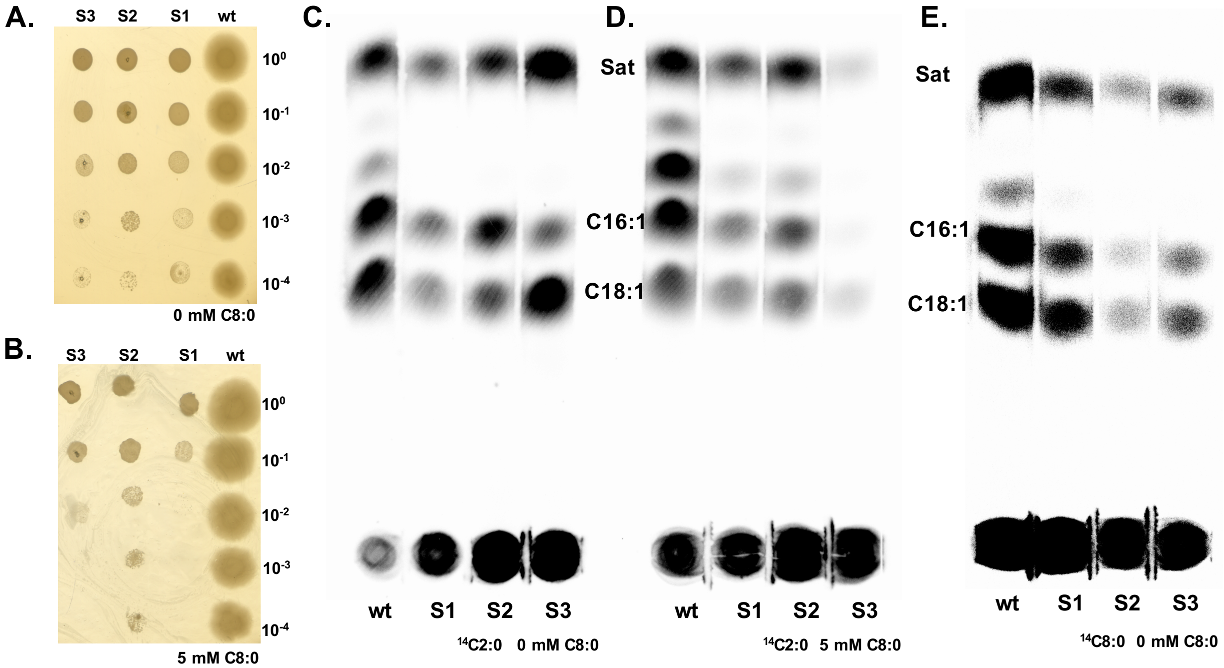

Figure S7. The phenotypes of the suppressor strains upon addition of octanoic acid (C8). **A**. Growth of the suppressor strains and the wild type strain on solid LB medium; **B**. growth of suppressor strains and the wild type strain on solid LB medium containing 5 mM C8; **C**. The phospholipid fatty acids of the wild type and suppressors were labeled with [1-^{14}C]acetate; **D**. The phospholipid fatty acids of the wild type and suppressors were labeled with [1-^{14}C]acetate with 5 mM C8; **E.** The phospholipid fatty acids of the wild type and suppressors were labeled with [1-^{14}C]octanoic acid.

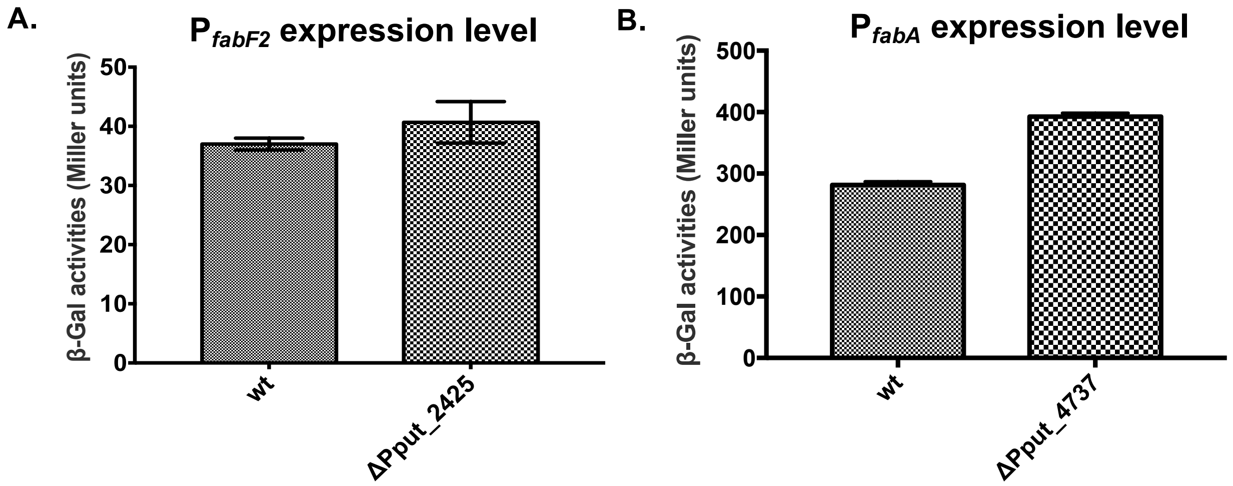

Figure S8. The expression levels of P*fabF2* and P*fabA*. A. β-Galactosidase activity of P*fabF2* in wild type and ΔPput_2425; **B**. β-Galactosidase activity of *PfabA* in wild type and ΔPput_4737.

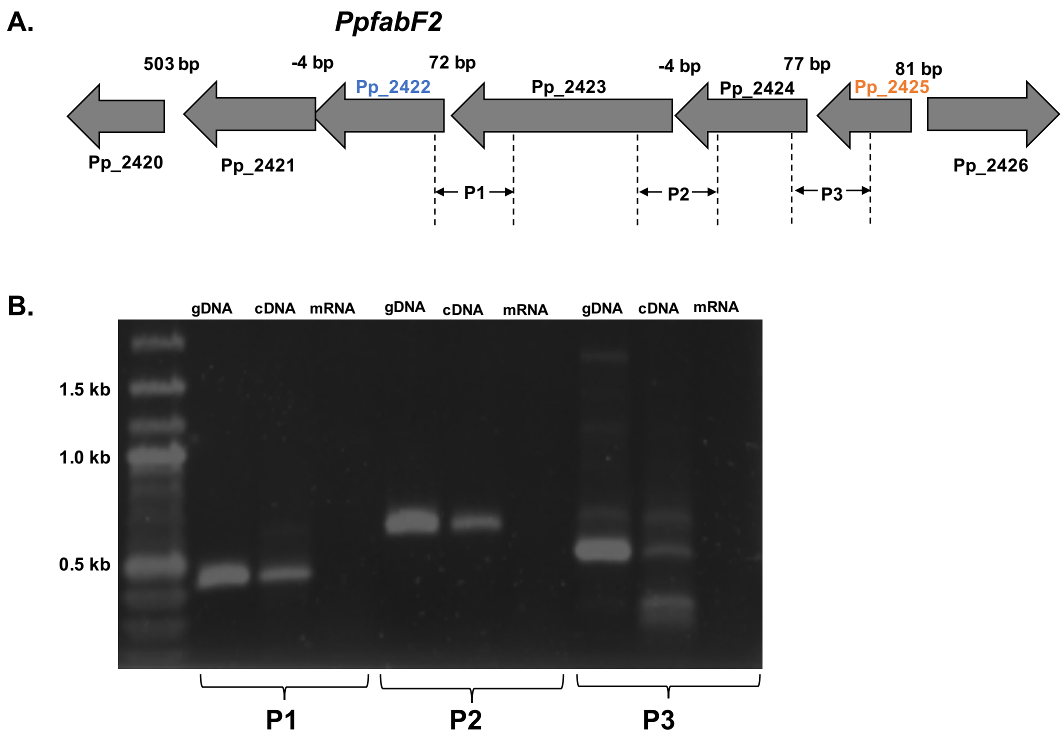

Figure S9. Transcriptional analysis of *fabF2* gene cluster.

A. Schematic diagram of the gene cluster in which *fabF2* is located; **B**. RT-PCR detection of the *fabF2* gene cluster mRNA showing that the cluster genes are cotranscribed and hence constitute an operon.

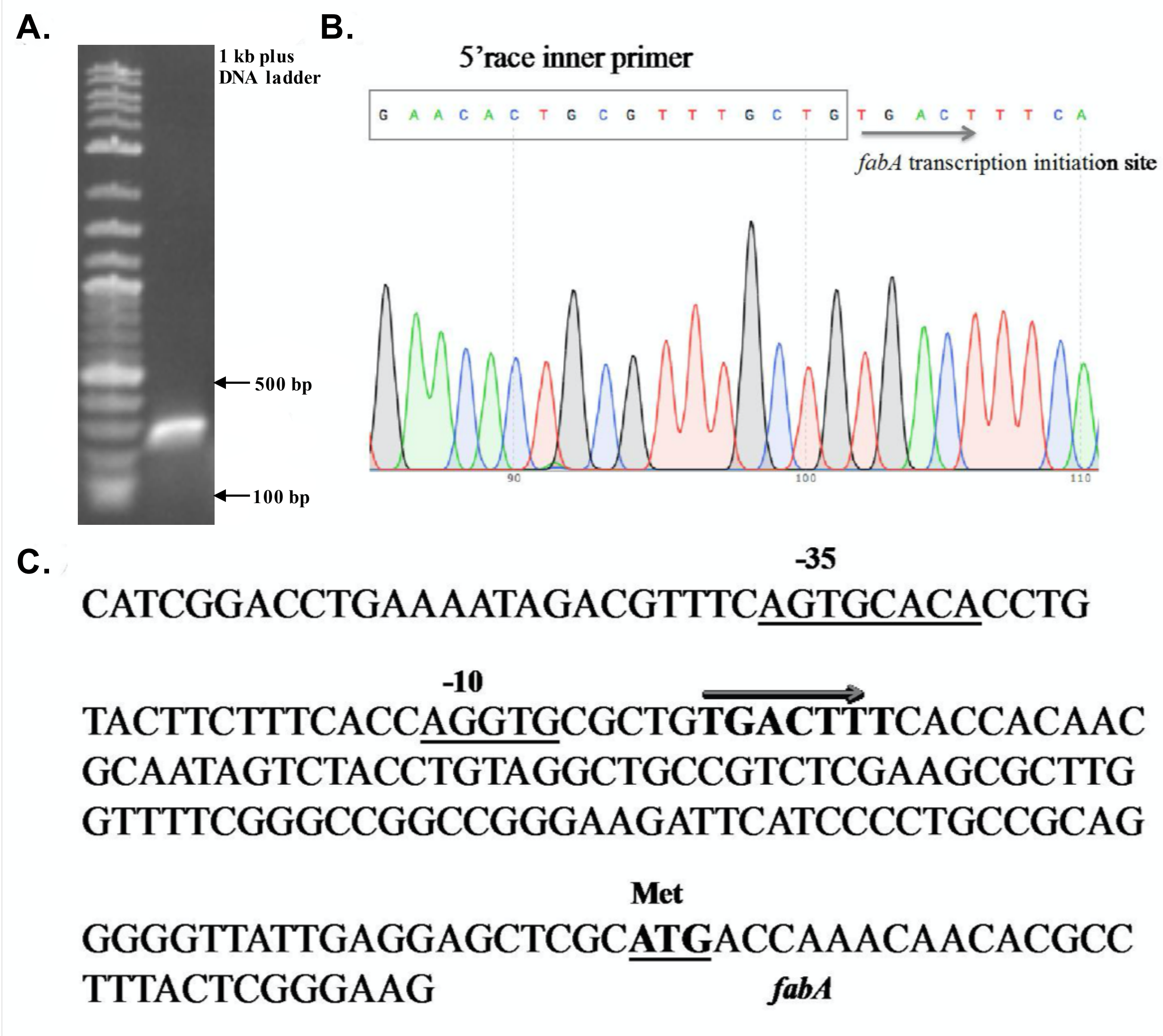

Figure S10. Analysis and confirmation of the transcription initiation site of *P. putida* F1 *fabA*. **A**. PCR fragments used for obtaining the *fabA* transcription start; **B**. sequence readout of the transcription start analysis; **C**. The location of the *fabA* transcription initiation site relative to the coding sequence.

A.

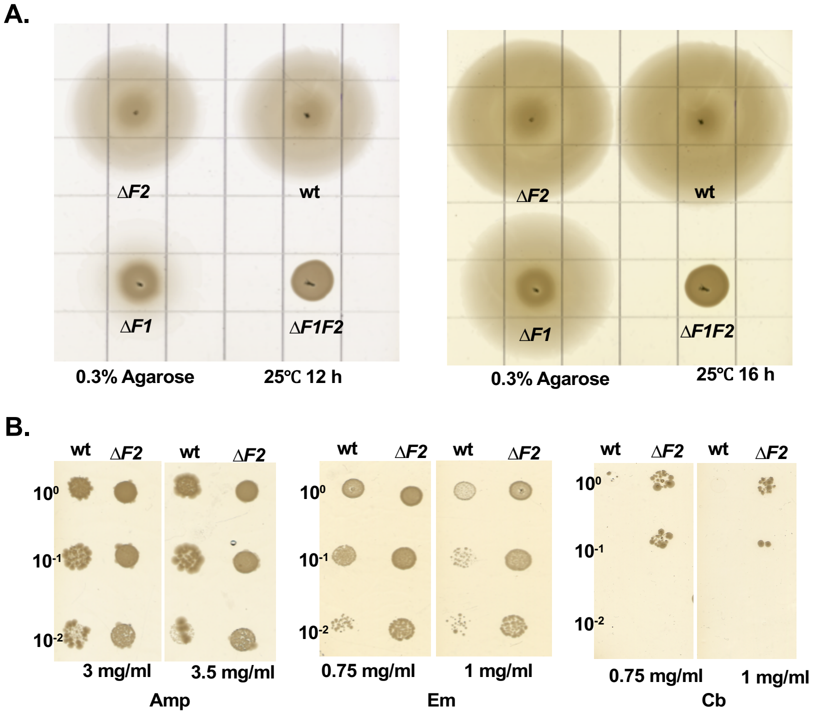

Figure S11. Analysis of the swimming motility and antibiotic resistance of Δ*fabF* strains. A. Swimming motility migration of Δ*fabF* strains, the migration of Δ*fabF1* (Δ*F1*) showed greatly decreased motility whereas the Δ*fabF1* Δ*fabF2* (Δ*F1F2*) almost totally lost swimming ability. The Δ*fabF2 (*Δ*F2*) showed no change in mobility; **B**. The Δ*fabF2 (*Δ*F2*) strain showed increased the resistance to ampicillin (Amp), erythromycin (Em) and carbenicillin (Cb).

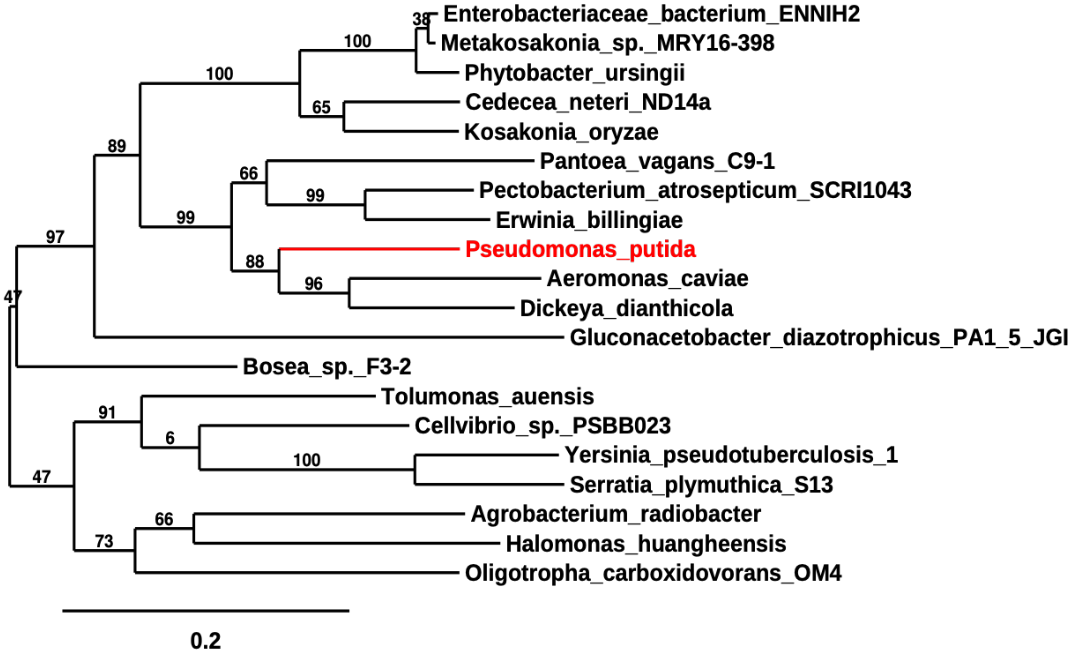

Figure S12. Phylogenetic tree of bacterial FabF2 proteins. The phylogenetic tree was generated by the website http://www.phylogeny.fr/simple_phylogeny.cgi?tab_index=2.
